# Arabidopsis MAPKKK δ-1 is required for full immunity against bacterial and fungal infection

**DOI:** 10.1093/jxb/erz556

**Published:** 2019-12-17

**Authors:** Tomoya Asano, Thi Hang-Ni Nguyen, Michiko Yasuda, Yasir Sidiq, Kohji Nishimura, Hideo Nakashita, Takumi Nishiuchi

**Affiliations:** 1 Institute for Gene Research, Advanced Science Research Center, Kanazawa University, Takaramachi, Kanazawa, Ishikawa, Japan; 2 Division of Life Science, Graduate School of Natural Science and Technology, Kanazawa University, Kanazawa, Ishikawa, Japan; 3 Plant Acquired Immunity Research Unit, RIKEN, 2-1 Hirosawa, Wako, Saitama, Japan; 4 Institute of Agricultural and Life Sciences, Academic Assembly, Shimane University, Matsue, Shimane, Japan; 5 Bielefeld University, Germany

**Keywords:** Disease resistance, *Fusarium*, immune response, MAPK cascade, MAPKKK, proteomics, protein phosphorylation, Raf kinase

## Abstract

The genome of Arabidopsis encodes more than 60 mitogen-activated protein kinase kinase (MAPKK) kinases (MAPKKKs); however, the functions of most MAPKKKs and their downstream MAPKKs are largely unknown. Here, MAPKKK δ-1 (MKD1), a novel Raf-like MAPKKK, was isolated from Arabidopsis as a subunit of a complex including the transcription factor AtNFXL1, which is involved in the trichothecene phytotoxin response and in disease resistance against the bacterial pathogen *Pseudomonas syringae* pv. tomato DC3000 (*Pst*DC3000). A MKD1-dependent cascade positively regulates disease resistance against *Pst*DC3000 and the trichothecene mycotoxin-producing fungal pathogen *Fusarium sporotrichioides*. *MKD1* expression was induced by trichothecenes derived from *Fusarium* species. MKD1 directly interacted with MKK1 and MKK5 *in vivo*, and phosphorylated MKK1 and MKK5 *in vitro*. Correspondingly, *mkk1* mutants and *MKK5RNAi* transgenic plants showed enhanced susceptibility to *F. sporotrichioides.* MKD1 was required for full activation of two MAPKs (MPK3 and MPK6) by the T-2 toxin and flg22. Finally, quantitative phosphoproteomics suggested that an MKD1-dependent cascade controlled phosphorylation of a disease resistance protein, SUMO, and a mycotoxin-detoxifying enzyme. Our findings suggest that the MKD1–MKK1/MKK5–MPK3/MPK6-dependent signaling cascade is involved in the full immune responses against both bacterial and fungal infection.

## Teaser

MKD1, a novel mitogen activated protein kinase kinase kinase, is part of the transcription factor AtNFXL1 complex and required for full immune responses against both bacterial and fungal infection.

## Introduction

Mitogen-activated protein kinase (MAPK) cascades are important in signal transduction during adaptation to biotic and abiotic stresses in all eukaryotes. The genome of Arabidopsis encodes 20 MAPKs, 10 MAPK kinases (MAPKKs), and 60–80 MAPKK kinases (MAPKKKs) (Ichimura *et al.*, 2002; Samaj *et al.*, 2004; Colcombet and Hirt, 2008; Rao *et al.*, 2010). Although several Arabidopsis MAPKs and MAPKKs are known to regulate disease resistance against phytopathogens, only a few MAPKKKs have been reported to be involved in this resistance (Asai *et al.*, 2002). Arabidopsis MAPKKK (MEKK1)-dependent MAPK cascades (MEKK1–MKK1/MKK2–MPK4) positively regulate innate immune responses against both the bacterial pathogen *Pseudomonas syringae* pv. tomato DC3000 (*Pst*DC3000) and the fungal pathogen *Botrytis cinerea* (Asai *et al.*, 2002; Menke *et al.*, 2004; Mészáros *et al.*, 2006; Brader *et al.*, 2007; Dóczi *et al.*, 2007; Suarez-Rodriguez *et al.*, 2007; Gao *et al.*, 2008; Kawasaki *et al.*, 2017; Thulasi Devendrakumar *et al.*, 2018). The Arabidopsis MAPKKK Enhanced Disease Resistance 1 (EDR1) belongs to the 10-member B3 subgroup of Arabidopsis Raf-like MAPKKKs (Ichimura *et al.*, 2002). EDR1 negatively regulates disease resistance against *Pst*DC3000 and the fungal phytopathogens *Erysiphe cichoracearum* and *Golovinomyces cichoracearum* (Frye and Innes, 1998; Frye *et al.*, 2001; Zhao *et al.*, 2014). EDR1 interacts with MKK4 and MKK5 and negatively regulates MPK3 and MPK6 activities (Zhao *et al.*, 2014). Another Raf-like MAPKKK, CTR1, suppresses the ethylene response by inactivating the MKK9–MPK3/MPK6 cascade (Colcombet and Hirt, 2008).

*Pst*DC3000 is a bacterial pathogen; both its compatible and its incompatible interactions with Arabidopsis are well-studied (Mansfield *et al.*, 2012). Furthermore, its flagellin flg22 peptide is frequently used to investigate pathogen-associated molecular pattern (PAMP)-induced plant immune responses (Mészáros *et al.*, 2006; Gao *et al.*, 2008; Bethke *et al.*, 2009). *Fusarium* species are fungal pathogens that produce trichothecene mycotoxins and are responsible for *Fusarium* head blight, a serious disease in crops such as wheat, barley, and maize (Eudes *et al.*, 2001; Xu *et al.*, 2007; Walter *et al.*, 2010). Arabidopsis is also susceptible to deoxynivalenol (DON)-producing *Fusarium* species such as *F. graminearum* (Chen *et al.*, 2009). We described the defense response of Arabidopsis against the mycotoxin (T-2 toxin)-producing *F. sporotrichioides* (Asano *et al.*, 2012).

In a previous study, we reported that some trichothecenes, such as the T-2 toxin, also act as elicitors and induce prolonged activation of certain MAPKs in Arabidopsis (Nishiuchi *et al.*, 2006). Subsequently, we isolated the Arabidopsis transcription factor gene, *AtNFXL1*, as a trichothecene-inducible gene, and found that the *atnfxl1* mutant shows hypersensitivity to trichothecenes and enhanced disease resistance against *Pst*DC3000 (Asano *et al.*, 2008). AtNFXL1 negatively regulates these responses by way of SA-dependent signaling (Asano *et al.*, 2008). To study the molecular function of AtNFXL1, we isolated a protein complex containing AtNFXL1 from T-2 toxin-treated plants. Here we report that MAPKKK δ-1 (MKD1), a novel Raf-like MAPKKK, forms part of the AtNFXL1-containing protein complex. MKD1 positively regulated the phytotoxin response as well as disease resistance against *Pst*DC3000 and *F. sporotrichioides*. Furthermore, a MKD1-dependent MAPK signaling cascade was discovered.

## Materials and methods

### Growth conditions of WT and mutant plants

Plants were grown at 22 °C under long-day conditions (16 h light–8 h dark) in a growth chamber. The *mkd1* (SALK_048985), *mkk1* (SALK_140054), *mkk2* (GABI_835B02), and *mpk6* mutants (SALK_127507) were obtained from the Arabidopsis Biological Resource Center (Ohio State University, Columbus, OH, USA). For an expression study, the plants were grown on Murashige and Skoog (MS) agar medium for 10 d and then were transferred to MS agar medium containing 0.5 µM T-2 toxin, 2.5 µM diacetoxyscirpenol (DAS), 10 µM DON, or 10 µM flg22. For phytotoxin sensitivity of some mutants, the plants were grown on MS agar medium containing 0.5 µM T-2 toxin.

### Fungal and bacterial inoculation assays

The *F. sporotrichioides* inoculation assay was performed as previously described (Asano *et al.*, 2012). WT, *mkd1*, *mkk1*, *mkk2*, and *MKK5RNAi* transgenic plants were grown on soil for about 28 d. After inoculation, plants were incubated under about 100% relative humidity for 2 d, at 22 °C, and a 16/8 h light–dark cycle. The *Pst*DC3000 inoculation assay was performed as previously described (Yasuda *et al.*, 2003).

### Preparation of His–AtNFXL1ΔNΔZn protein in *E. coli* and anti-AtNFXL1C antibody

The *AtNFXL1*Δ*N*Δ*Zn* fragment (2341–3567 bp) was amplified by PCR from cDNA using specific primers (see Supplementary Table S1 at *JXB* online). The amplified fragment of *AtNFXL1*Δ*N*Δ*Zn* was cloned into the *Nde*I and *Sal*I sites of the pET-29a vector (Merck KGaA). The plasmids were transformed into *E. coli* BL21-CodonPlus (DE3)-RIL (Agilent Technologies). The 6×Histidine (His) tag-labelled AtNFXL1ΔNΔZn protein (His–AtNFXL1ΔNΔZn protein) was purified using a Ni Sepharose High Performance column (GE Healthcare). SDS-PAGE and immunoblotting were carried out as previously described (Asano *et al.*, 2004). The anti-AtNFXL1C antibody was generated in rabbit and purified using antigen (His–AtNFXL1ΔNΔZn protein)-coupled HiTrap^TM^ NHS (*N*-hydroxysuccinimide)-activated HP (high performance; GE Healthcare). Then, an AtNFXL1-containing protein complex was purified using anti-AtNFXL1C antibody coupled to HiTrap^TM^ NHS-activated HP.

### Purification of the AtNFXL1-containing protein complex and identification of subunits

To purify the complex containing the AtNFXL1 protein, we used 5 g tissue from WT and *atnfxl1* mutant plants treated with 0.5 µM T-2 toxin. Tissues were ground to a fine powder in liquid nitrogen with a pestle and lysed with extraction buffer (10 mM HEPES–KOH buffer (pH 8.0) containing 1% Triton X-100 and a protease-inhibitor cocktail (Roche Diagnostics K.K.)). Following centrifugation, the supernatants were mixed with 5 volumes of extraction buffer. The AtNFXL1 protein complex was purified using an anti-AtNFXL1C antibody-coupled HiTrap^TM^ NHS-activated HP column. The complexes were eluted with 0.1 M glycine–HCl (pH 2.3). The resulting elutions were mixed with a 1/20 volume of 1 M Tris buffer and subjected to SDS-PAGE. Silver staining was performed using a Silver Stain MS Kit (Wako pure Chemical Industries) according to the manufacturer’s standard protocol. WT-specific bands were excised from the gel with a scalpel, cut into small pieces, and de-stained according to the manufacturer’s standard protocol. In-gel digestion by trypsin was performed as previously described (Asano and Nishiuchi, 2011). The peptides were purified using ZipTipC18 columns (Millipore) according to the manufacturer’s protocol and mixed with α-cyano-4-hydroxycinnamic acid (α-CHCA) on the sample plate for matrix-assisted laser desorption/ionization (MALDI) time of flight (TOF) mass spectrometer (Voyager DE-STR; AB Sciex). In addition, the data for the obtained peak were analysed by searching a protein sequence database (ProFound^TM^ database at Rockefeller University).

### **Pull down assay with His–AtNFXL1**Δ**N**Δ**Zn and biotin–MKD1**

The *MKD1* gene was amplified by RT-PCR using specific primers (see Supplementary Table S1). The amplified PCR products were introduced into the *Eco*RI and *Sal*I sites of the pTNT vector (Promega). Biotin-labelled proteins were synthesized using the TNT^®^ Coupled Rabbit Reticulocyte Lysate System (Promega). The *in vitro* transcription/translation was performed according to the protocol of the TNT^®^ Quick Coupled Transcription/Translation system. His–AtNFXL1ΔNΔZn protein and biotin–MKD1 protein were used for the pull down assay with Ni Sepharose High Performance columns. The biotin–MKD1 protein was detected with the Transcend™ Non-Radioactive Translation Detection Systems (Promega).

### Bimolecular fluorescence complementation analysis of interaction between MKD1 and MKKs in onion epidermis

All plasmids used for bimolecular fluorescence complementation (BiFC) analysis in onion (*Allium cepa*) epidermis were constructed using the Gateway cloning method according to the manufacturer’s instructions (Thermo Fisher Scientific). The coding regions of *MKD1* and *MKKs* were amplified by PCR using specific primers (see Supplementary Table S1). The amplified DNA fragments were cloned into pENTR/D-TOPO (Thermo Fisher Scientific) by a BP reaction (attB×attP→attL×attR) for the construction of the corresponding entry clones. A series of modified V10-BiFC destination vectors were generated as follows (Nishimura *et al.*, 2015). Briefly, these BiFC vectors were made by introducing the *Xba*I–*Sac*I fragment of each V10-BiFC vector containing the Gateway cassette together with split monomeric Venus fragment into the linearized pGWB402 vector harboring *Xba*I and *Sac*I ends to make the Gateway-compatible binary V10-BiFC vectors such as pB4nVGW3, pB4cVGW, pB4GWnV3, and pB4GWcV (Nakagawa *et al.*, 2007; Nishimura *et al.*, 2015). The complete nucleotide sequences of the binary V10-BiFC vectors were registered in GenBank/EMBL/DDBJ as AP019390 (pB4nVGW3), AP019391 (pB4cVGW), AP019392 (pB4GWnV3), and AP019393 (pB4GWcV). The resultant entry clones were subjected to an LR (attL×attR→attB×attP) reaction with the binary V10-BiFC destination vectors for generation of the corresponding expression vectors, which were used for a protein–protein interaction analysis by the transient BiFC system through biolistic bombardment in onion epidermal cells. One microgram of a pair of binary V10-BiFC expression vectors and 1 µg of pUGW45 as an internal reference were used for coating on tungsten particles (Nakagawa *et al.*, 2007). A BiFC assay was performed as previously reported (Nishimura *et al.*, 2015).

### BiFC analysis using Arabidopsis transgenic plants

For BiFC analysis using Arabidopsis transgenic plants, the coding regions of the *AtNFXL1*, *MKKs*, and *MKD1* genes were amplified using specific primers (see Supplementary Table S1). The *AtNFXL1* gene was introduced into the pB5NY0 and pB5NY2 plasmids (gifts from S. Mano, National Institute for Basic Biology) by Gateway technology (Hanano and Goto, 2011). *MKD1* was introduced into pB5CY0. *MKK1*, *MKK2*, and *MKK5* were introduced into B5NY0. The plasmids were transformed into WT plants by *in planta* transformation. YFPN–AtNFXL1, AtNFXL1–YFPN, YFPN–MKK1, YFPN–MKK2, and YFPN–MKK5 transgenic plants were artificially pollinated with pollen from YFPC–MKD1 plants. Plants were grown on MS medium for 10 d. The yellow fluorescent protein (YFP) signal was visualized using an Olympus microscope (model BX50) with a DP71 camera system using a built-in BX-FLA epifluorescence unit.

### Generation of other transgenic plants

For generation of PMKD1:GUS plants, the *MKD1* promoter region was amplified by PCR using specific primers (see Supplementary Table S1), and was then introduced into the *Hin*dIII and *Xba*I sites of the pBI121 vector (Asano *et al.*, 2008). For complementation tests, the coding region of *MKD1* with 5′ flanking region (from −962 to 2369 bp) was amplified from Arabidopsis genomic DNA. The resulting fragment was introduced into *Hin*dIII and *Sac*I sites (blunt ended) of the pSMAH621 plasmid. To generate transgenic plants containing green fluorescent protein (GFP)-fused MKD1, MKK1, MKK5, and MPK6, we amplified the entire coding regions of fragments from WT cDNA by using specific PCR primers (Supplementary Table S1). *MKD1*, *MKK1*, *MKK5*, and *MPK6* fragments were inserted into the pH7WGF2.0 plasmid using Gateway technology. The *MKK5* region for RNAi was amplified from WT cDNA by PCR with specific primers (Supplementary Table S1). The resulting plasmids were introduced into the pANDA plasmid using Gateway technology (Miki and Shimamoto, 2004). The plasmids were then transformed into WT plants by *in planta* transformation.

### RT-qPCR

RT-qPCR was performed as previously described (Asano *et al.*, 2008). *MKD1*, *ACT2/8*, *PR1*, and *PDF1.2* were amplified from cDNA using specific primers (see Supplementary Table S1). The mRNA levels were normalized to those of *ACTIN2* and *ACTIN8* as reference gene.

### Microscopic observation

PMKD1:GUS transgenic plants were fixed in 90% acetone at −20 °C and then incubated in a β-glucuronidase (GUS) staining buffer at 37 °C for 2 h (Koizumi *et al.*, 2000). Plastic sections were prepared with a Technovit 7100 system (Heraeus Kulzer). The hyphae of *F. sporotrichioides* were stained with Trypan blue solution (Mackey *et al.*, 2002).

### Yeast two-hybrid analysis of MKD1 and AtNFXL1

The *MKD1* and *AtNFXL1* coding regions were amplified by PCR using specific primers (see Supplementary Table S1). To perform yeast two-hybrid analysis, amplified partial fragments derived from *AtNFXL1* were subcloned into a pGBKT7 vector (Clontech). The amplified *MKD1* PCR products were subcloned into a pGADT7 vector (Clontech), and the resulting plasmids were transformed into yeast strain Y190. To check proper yeast cell growth, transgenic yeast cells were streaked on SD medium without tryptophan and leucine (SD−WL). The SD medium without His, Trp, and Leu (SD−HWL) indicates positive results in the yeast two-hybrid assay. The addition of 3-amino-1,2,4-triazole (3-AT) was used to perform the yeast two-hybrid assay in more stringent conditions, indicating the strength of protein–protein interaction.

### Yeast two-hybrid analysis of MKD1, MKKs, and MPK6

The coding regions of *MKD1*, *MKKs*, and *MPKs* were amplified by PCR using specific primers (see Supplementary Table S1). Amplified *MKK* fragments were cloned into *Sma*I and *Pst*I sites of the pGBKT7 plasmid (Clontech), and amplified *MKD1* and *MPKs* fragments were cloned into *Sma*I and *Xho*I sites of the pGADT7 plasmid (Clontech). The resulting plasmids were transformed into the yeast strain Y190. β-Galactosidase activity showing the strength of protein–protein interaction was measured in diploids according to the Yeast Protocols Handbook (Clontech; http://www.clontech.com).

### **Preparation of** Δ**MKD1–His protein, MKK1, MKK2, and MKK5 proteins**

The sequence coding for 6×His tag-labelled ΔMKD1 (ΔMKD1) was amplified by RT-PCR using specific primers (see Supplementary Table S1). The amplified PCR products were introduced into *Sgf*I and *Pme*I sites of the pF3KWG (BYDV) Flexi plasmid (Promega). The ΔMKD1 protein was synthesized using the TNT SP6 High-Yield Wheat Germ Protein Expression System (Promega). *In vitro* transcription/translation was performed according to the manufacturer’s instructions. The ΔMKD1 protein was purified using a Ni Sepharose High-Performance column (GE Healthcare). *MKK1*, *MKK2*, and *MKK5* were amplified from cDNA by PCR using specific primers (Supplementary Table S1). Amplified fragments of *MKK1*, *MKK2*, and *MKK5* were cloned into *Sma*I and *Not*I (blunt ended) sites of the pGEX6p-1 plasmid (GE Healthcare). *MKK1*, *MKK2*, and *MKK5* plasmids were transformed into *E. coli* BL21-CodonPlus (DE3)-RIL (Agilent Technologies). The recombinant proteins glutathione *S*-transferase (GST)–MKK1, GST–MKK2, and GST–MKK5 were digested by PreScission Protease (GE Healthcare). The resulting MKK1, MKK2, and MKK5 proteins were purified using a glutathione Sepharose 4 Fast Flow column (GE Healthcare).

### Kinase assay

An *in vitro* ΔMKD1 kinase assay using MKKs was performed as previously described (Nishihama *et al.*, 2001). The assay used 200 ng protein. An in-gel kinase assay was performed using 20 µg total proteins as described elsewhere (Nishiuchi *et al.*, 2006). An immunoprecipitation kinase assay was performed using anti-MPK4 (a gift from Y. Machida, Nagoya University) and anti-MPK6 (Sigma-Aldrich) antibodies as previously described (Nishiuchi *et al.*, 2006). The phosphorylation sites were determined by LC-MALDI analysis. The phosphorylated MKK1 and MKK5 proteins were digested by chymotrypsin (Roche). The peptides were analysed using a 4800 Plus MALDI TOF/TOF^TM^ Analyzer (AB Sciex). tandem mass spectrometry (MS/MS) data were evaluated by comparing amino acid substitutions and modifications against the NCBI database using the Paragon algorithm (Shilov *et al.*, 2007) of the ProteinPilot^™^ v2.0 software (AB Sciex).

### iTRAQ analysis

WT and *mkd1* mutant plants were grown on MS-0 medium for 16 d. Then, WT and *mkd1* mutant plants were treated on MS agar medium containing 1 µM T-2 toxin for 3 h. Roots of WT and *mkd1* mutant plants were collected, and phosphoproteins were purified using the Pro-Q^®^ Diamond Phosphoprotein Enrichment Kit (Invitrogen; Asano and Nishiuchi, 2011). Total proteins and phosphoproteins were stained with SYPRO Ruby Protein Gel Stain and Pro-Q^®^ Diamond Phosphoprotein Gel Stain, respectively. WT and *mkd1* mutant proteins (100 µg each) were labelled using the iTRAQ^®^ Reagents according to the manufacturer’s instructions (AB Sciex). Peptides derived from WT and *mkd1* mutant were labelled with tags 114 and 117, respectively. The labelled peptides were analysed using a 4800 Plus MALDI TOF/TOF^TM^ Analyzer (AB Sciex). MS/MS data were evaluated by comparing amino acid substitutions and modifications against the NCBI database using the Paragon algorithm (Shilov *et al.*, 2007) of the ProteinPilot^™^ v2.0 software (AB Sciex).

## Results

### **Raf-like MAPKKK** δ**-1 (MKD1) protein is a subunit of the AtNFXL1-containing protein complex**

**Figure 1.**
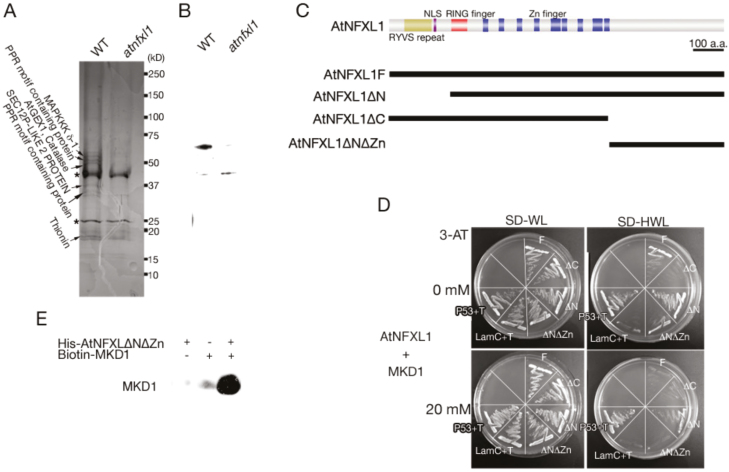
Protein–protein interaction between MKDI and AtNFXL1. (A) SDS-PAGE of the AtNFXL1 protein-containing complex purified from T-2 toxin-treated WT and *atnfxll* mutant plants using an anti-AtNFXL1C antibody column. Designations on the left side indicate identified subunits specifically observed in WT. Asterisks indicate non-specific proteins. (B) Western blot analysis of purified AtNFXL1-containing complex using anti-AtNFXL1C antibody. (C) Schematic diagram of full length and partial AtNFXL1 using yeast two-hybrid analysis. (D) The interaction between AtNFXL1 and MKDI was investigated by yeast two-hybrid analysis. The concentrations of 3-amino-1,2,4-triazole (3-AT) are shown on the left. P53+T and LamC+T represent the positive and negative controls, respectively. Similar results were obtained in three independent experiments. (E) The binding of AtNFXL1 protein to the MKD1 protein was examined by pull-down assays. His epitope-tagged AtNFXL1DNDZn protein was applied to a Ni Sepharose High Performance column. Biotin–MKD1 was detected by Transcend™ Non -Radioactive Translation Detection Systems.

We purified the AtNFXL1-containing complex to study the molecular function of this protein. Using an anti-AtNFXL1 C-terminal antibody linked to an affinity column, the AtNFXL1-containing protein complex could be isolated from preparations of wild type (WT) and *atnfxl1* mutant plants. When purified complexes were subjected to SDS-PAGE, six subunit proteins were specifically observed in the WT. These bands were excised and were digested by trypsin, and then were identified by MALDI-TOF as shown in Fig. 1A. Among them, we focused on the novel MAPKKK, the MKD1 protein (At5g11850) that was isolated as one of the subunits of the AtNFXL1-containing protein complex (Fig. 1A, B). The interaction of MKD1 with the C-terminal region of AtNFXL1 was comfirmed by yeast two-hybrid analysis (Fig. 1C, D) and immunoprecipitation assays (Fig. 1E). In addition, the zinc finger domain of the AtNFXL1 protein also affected the interaction with the MKD1 protein (Fig. 1C, D). BiFC analysis showed that MKD1 interacted with AtNFXL1 in the cytoplasm and nuclei (see Supplementary Fig. S1). These results suggested that MKD1 could be involved in *AtNFXL1*-related phytopathogen resistance and phytotoxin responses.

The MKD1 protein has a C-terminal kinase domain and an N-terminal putative regulatory domain (see Supplementary Fig. S2A). The amino acid sequence of MKD1 is similar to those of Raf-like MAPKKKs such as EDR1 (Frye *et al.*, 2001) and CTR1 (Kieber *et al.*, 1993; Supplementary Fig. S2A, B). The kinase domain is highly conserved among these Raf-like MAPKKKs (Supplementary Fig. S2C).

### The expression pattern of MKD1

**Figure 2.**
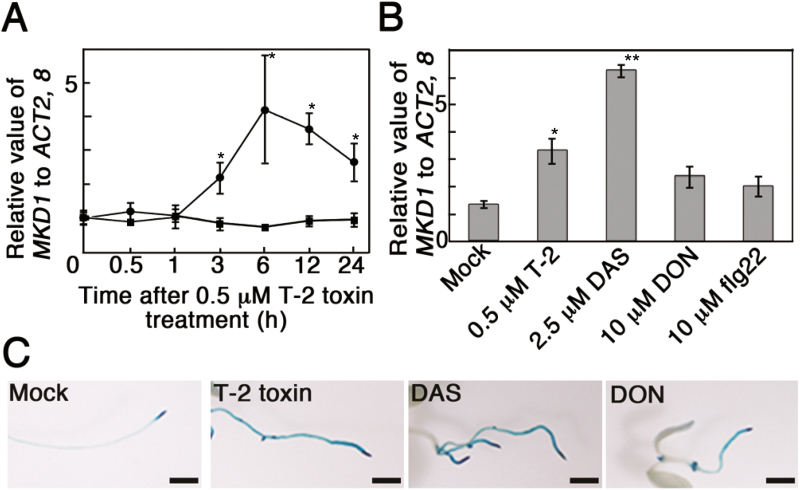
Induction of *MKD1* mRNA by trichothecenes. (A) Expression levels of *MKD1* after T-2 toxin treatment were analysed by RT-qPCR. Circles and squares show the data from T-2 toxin- and mock-treated samples, respectively. Data points represent the mean ±SD (*n*=3). *P<0.05, based on Student’s *t*-test. (B) Expression levels of *MKD1* 6 h after DAS, DON, or flg22 treatment were analysed by RT-qPCR. Data points represent means ±SD (*n*=5). *P<0.05, **P<0.01, based on Student’s *t*-test. Similar results were obtained in three independent experiments. (C) Representative photos of GUS staining in PMKD1:GUS plants grown on MS agar medium with or without trichothecene (T-2 toxin, DAS, or DON). Scale bars: 1 mm. Similar results were obtained for more than 10 transgenic plants with each trichothecene treatment. (This figure is available in color at *JXB* online.)

To investigate the expression pattern of *MKD1*, we introduced MKD1 promoter:β-glucuronidase (GUS) fusion genes into Arabidopsis. In the *MKD1* promoter–GUS plants (PMKD1:GUS), GUS staining was observed mainly in young leaves, shoot apices, vascular bundles, and guard cells (see Supplementary Fig. S3). The *MKD1* mRNA level was transiently increased by the T-2 toxin (Fig. 2A). *MKD1* expression in seedlings was significantly increased by treatment with type A trichothecene phytotoxins (T-2 toxin, diacetoxyscirpenol (DAS); Fig. 2B, C). Trichothecene-inducible expression was observed predominantly in roots (Fig. 2C). Similar expression patterns have been observed for the *AtNFXL1* gene (Asano *et al.*, 2008). These results suggested that the biological function of MKD1 is related to that of the *AtNFXL1* gene.

### The *mkd1* mutant showed enhanced susceptibility to *Pst*DC3000 and *F. sporotrichioides*

**Figure 3.**
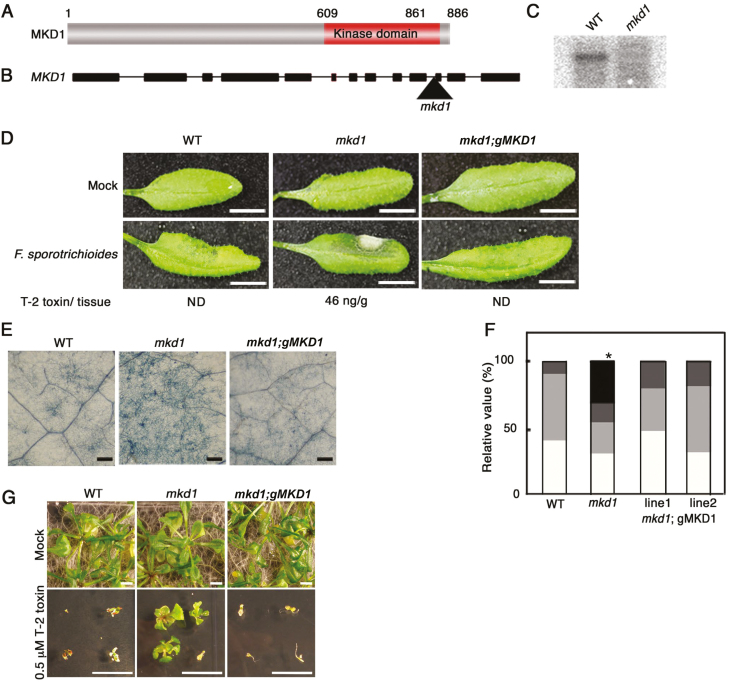
MKD1 is involved in disease resistance against bacterial and fungal phytopathogens. (A) Schematic structure of the MKD1 protein. (B) Position of the T-DNA insertion in the *mkd1* mutant. Boxes show exons. Triangle indicates the insertion position of the T-DNA. (C) RNA gel blot analysis of *MKD1* mRNA in WT and the *mkd1* mutant. (D) Representative images of WT, the *mkd1* mutant, and the complementation line (*mkd1*;*gMKD1* transgenic plants) 2 d after inoculation with *F. sporotrichioides* conidia. Scale bars: 1 cm. T-2 toxin/tissue indicates the concentration of T-2 toxin in leaves. ND: not detected. Similar results were obtained in three independent experiments. (E) Trypan blue staining of *F. sporotrichioides*-inoculated leaves after 2 d. Scale bars: 100 µm. (F) Relative values for the classification of disease symptoms in *F. sporotrichioides*-inoculated leaves (*n*=17–30). The bars show disease severity. White (class 1): normal, light gray (class 2): leaf turned black; dark gray (class 3): partial hyphae; black (class 4): expanded aerial hyphae. *P<0.05, based on Man–Whitney *U*-test. (G) Representative photos of WT and *mkd1* mutant; the complementation lines were grown on MS agar medium with or without 0.5 µM T-2 toxin for 2 weeks. Similar results were obtained in three independent experiments. Scale bars: 1 cm.

**Figure 4.**
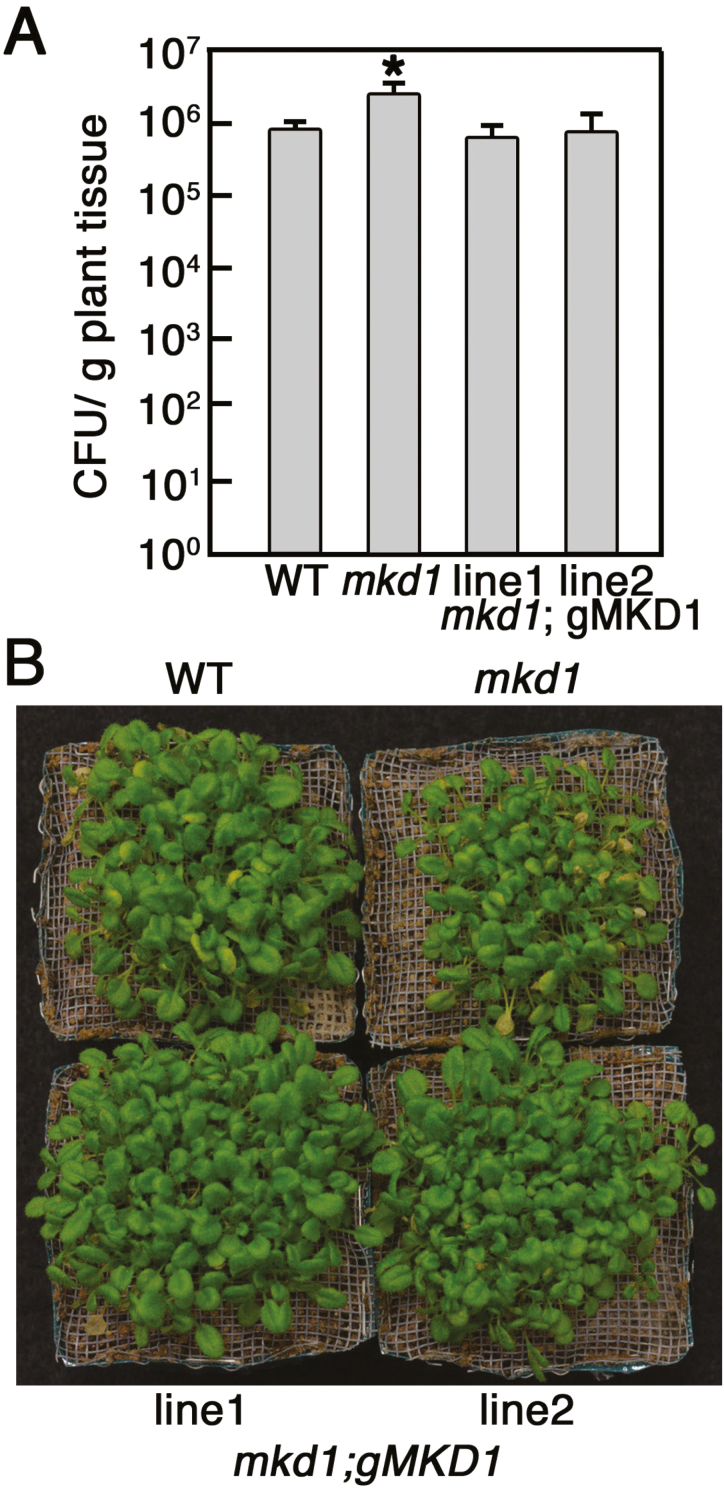
Enhanced susceptibility of *mkd1* mutant plants to the virulent pathogen *Pst*DC3000. (A) Colony forming units (CFU) are shown as means ±SD (*n*=6). A significant difference between WT and the *mkd1* mutant was observed in the number of CFU/g fresh weight (*P*<0.05, ANOVA). (B) Representative photos of WT and *mkd1* mutant; the complementation lines were inoculated with the *Pst*DC3000. Similar results were obtained in two independent experiments. (This figure is available in color at *JXB* online.)

Functional analysis of MKD1 was performed using a T-DNA-insertion *mkd1* mutant (Fig. 3B; Supplementary Fig. S2C). *MKD1* transcripts were not detected in the *mkd1* mutant, suggesting that *mkd1* is a null allele in these plants (Fig. 3C). We first examined the disease resistance against the virulent pathogen, *Pst*DC3000, and the T-2 toxin response. Compared with the WT, growth and morphology of *mkd1* mutant plants grown on Murashige and Skoog (MS) agar medium or soil showed no visible phenotypic change (Fig. 3D, G). However, the growth inhibition evoked by the T-2 toxin in plants grown on MS agar plates was reduced in the *mkd1* mutant compared with the WT (Fig. 3G). In addition, the *mkd1* mutant showed enhanced susceptibility to *Pst*DC3000 (Fig. 4). These results implied that MKD1 positively regulates *Pst*DC3000 resistance and T-2 toxin-inducible defense responses in Arabidopsis. Thus, MKD1 and AtNFXL1 have opposite roles in the phytotoxin response and resistance to *Pst*DC3000. As stated above, AtNFXL1 negatively regulated the salicylic acid (SA)-dependent signaling pathway in response to the T-2 toxin (Asano *et al.*, 2008), suggesting that MKD1 was also involved in the SA and jasmonic acid (JA)/ethylene (ET) signaling pathway. However, T-2 toxin-inducible expression of SA-inducible genes (*PR1*) (Makandar *et al.*, 2012) and of the JA/ET-inducible *PDF1.2* gene (Penninckx *et al.*, 1998) was not significantly different between WT and the *mkd1* mutant (see Supplementary Fig. S4).

We have reported that Arabidopsis ecotype Columbia-0 showed resistance to the T-2 toxin-producing *F. sporotrichioides* when conidia solutions (1×10^5^ conidia ml^−1^) were infiltrated into the abaxial side of rosette leaves (Asano *et al.*, 2012). However, the *mkd1* mutant inoculated with *F. sporotrichioides* allowed for increased hyphal growth and accumulation of the T-2 toxin (Fig. 3D–F). The susceptibility of the *mkd1* mutant was rescued by the introduction of genomic DNA of *MKD1* (Fig. 3D, F). Thus, the *MKD1* gene is required for disease resistance to the mycotoxigenic fungus *F. sporotrichioides* and is involved in the resistance to the bacterial pathogen *Pst*DC3000.

### MKD1 interacts with MKK1 and MKK5 *in vivo* and phosphorylates MKK1 and MKK5 *in vitro*

**Figure 5.**
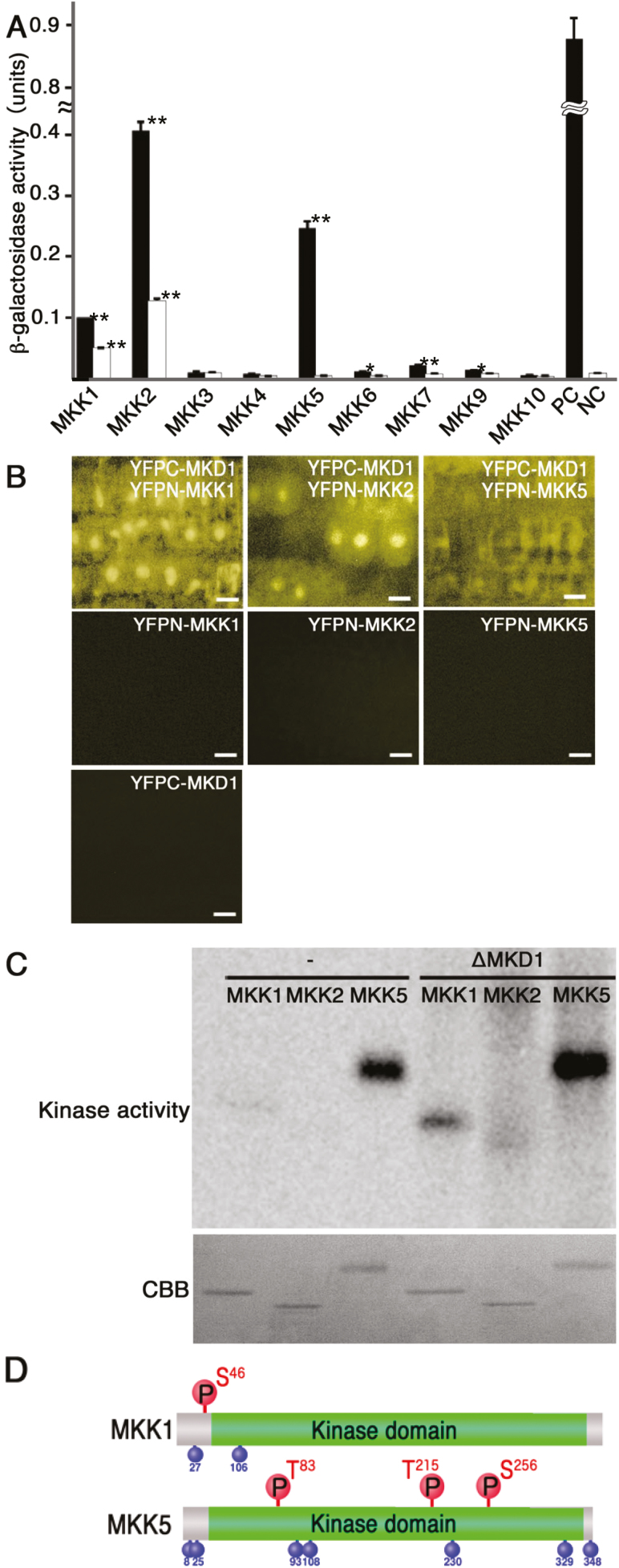
Downstream MKKs of the MKD1-dependent signaling cascade. (A) Protein–protein interactions between MKD1 and MKKs were examined by yeast two-hybrid analysis. The interactions were evaluated by β-galactosidase activity units per number of cells and incubation time. Black and white bars represent the values observed for the full-length MKD1 and for the kinase domain of MKD1, respectively. Results shown are means ±SD (*n*=3). *P<0.05, **P<0.01, based on Student’s *t*-test. Similar results were obtained in three independent experiments. (B) *In vivo* interactions of MKD1 with MKK1, MKK2, and MKK5 were examined by BiFC analysis. Images show the YFP signal in the root tip. Scale bars: 10 µm. Similar results were obtained in two independent experiment. (C) Phosphorylation of MKK1, MKK2, and MKK5 by constitutively active MKD1 (ΔMKD1) investigated by *in vitro* kinase assays. −, without ΔMKD1. (D) Phosphorylation sites on MKK1 and MKK5 targeted by MKD1. Phosphorylation sites targeted by MKD1 are shown above; autophosphorylation sites are shown below. S, serine; T, threonine. (This figure is available in color at *JXB* online.)

To identify downstream targets of MKD1, we performed yeast two-hybrid-based protein–protein interaction analyses of MKD1 and nine Arabidopsis MAPKKs (MKKs). Yeast carrying full-length MKD1 along with MKK1, MKK2, or MKK5, but not any other MKKs, showed increased β-galactosidase activity (Fig. 5A). As stated above, MKK1, MKK2, MKK4, and MKK5 are involved in the plant immune response. Therefore, the interaction between MKD1 and MKK1, MKK2, and MKK5 were also examined by the BiFC method in onion epidermis. Supplementary Fig. S5 suggests that MKD1 can also interact with MKK1, MKK2, and MKK5 in plant cells. In addition, weak BiFC signals were observed between MKD1 and MKK4. Based on these results, we further investigated the interaction between MKD1 and MKK1, MKK2, and MKK5.

Yeast expressing the kinase domain of MKD1 along with MKK1 or MKK2, but not MKK5, showed relatively weak β-galactosidase activity compared with yeast carrying the full-length MKD1 (Fig. 5A), indicating that the N-terminal region of MKD1 is required for the interaction with MKK5. The N-terminal region of MKD1 also affected its binding to MKK1 and MKK2. To verify these interactions, we conducted BiFC analyses using Arabidopsis transgenic plants. YFP signals were analysed in plants co-expressing YFPC–MKD1with YFPN–MKK1, YFPN–MKK2, or YFPN–MKK5 (Fig. 5B). YFP signals were observed in the cytoplasm and nuclei of plants carrying YFPC–MKD1 and YFPN–MKK1 or YFPC–MKD1 and YFPN–MKK2, indicating that interactions between MKD1 and MKK1 or MKK2 took place in the cytoplasm and nucleus (Fig. 5B). On the other hand, fluorescence signals of plants carrying YFPC–MKD1 and YFPN–MKK5 implied interactions between MKD1 and MKK5 in the cytoplasm.

Furthermore, to examine whether MKD1 directly phosphorylates MKK1, MKK2, or MKK5, we performed *in vitro* kinase assays using a constitutively active form of MKD1 (ΔMKD1) without the N-terminal regulatory region (Asai *et al.*, 2002) and full-length MKK1, MKK2, and MKK5. MKK1 and MKK5 showed autophosphorylation activities. Application of ΔMKD1 increased the phosphorylation of MKK1 and MKK5 (Fig. 5C). We attempted to identify the phosphorylation sites in the MKKs targeted by MKD1. LC-MALDI analysis revealed that the phosphorylation target sites for MKD1 in both MKK1 and MKK5 were different from the autophosphorylation target sites. ΔMKD1 phosphorylated Ser46 of MKK1 (Fig. 5D; Supplementary Table S2) and Thr83, Thr215, and Ser256 of MKK5 (Fig. 5D; Supplementary Table S2). In contrast, ΔMKD1 did not phosphorylate MKK2 (Fig. 5C) or MKK4 (Supplementary Fig. S6).

### The *mkk1* mutant and *MKK5RNAi* transgenic plants show susceptibility to *F. sporotrichioides*

**Figure 6.**
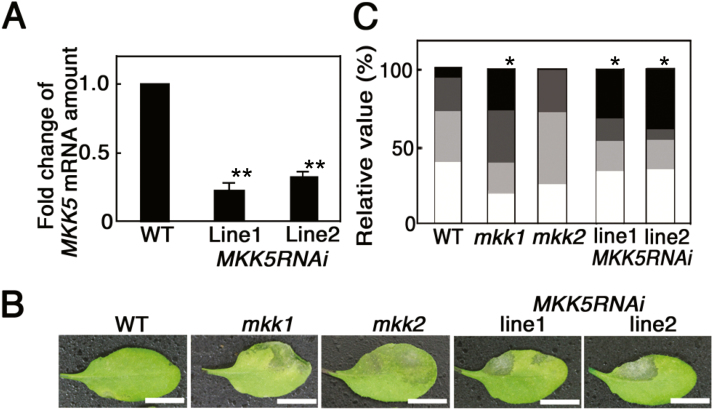
Resistance of *mkk1*, *mkk2*, and *MKK5RNAi* transgenic plants against *F. sporotrichioides*. (A) Suppression of *MKK5* mRNA in *MKK5RNAi* transgenic plants grown on MS medium containing 0.5 µM T-2 toxin. Amounts of *MKK5* mRNA were normalized against *ACTIN2*, *8*. *MKK5* mRNA levels in *MKK5RNAi* transgenic plants are represented as fold changes of the WT level (*n*=5).**P<0.01, based on Student’s *t*-test. Similar results were obtained in two independent experiment. (B) Representative images of WT, *mkk1*, *mkk2*, and *MKK5RNAi* leaves 2 d after inoculation with *F. sporotrichioides*. Similar results were obtained in three independent experiments. Scale bars: 1 cm. (C) Relative values for the classification of disease symptoms (*n*=17–30). Bars describe data as explained in Fig. 3F.*P<0.05, based on Man–Whitney *U*-test. (This figure is available in color at *JXB* online.)

We obtained the *mkk1* and *mkk2* T-DNA insertional mutants and generated *MKK5RNAi* transgenic plant lines to analyse the biological function of these genes. *MKK1* and *MKK2* mRNA in these plants was investigated by RT-PCR. *MKK1* and *MKK2* mRNAs were not detected in the corresponding mutant (see Supplementary Fig. S7), indicating that these mutants carry null alleles. *MKK5* mRNA levels were decreased by approximately one-fourth in *MKK5RNAi* plants compared with the WT (Fig. 6A). The *mkk1* mutant was previously reported to be susceptible to *Pst*DC3000 (Mészáros *et al.*, 2006), and *35S:MKK5* plants show enhanced resistance to *Pst*DC3000 (Asai *et al.*, 2002). Thus, both MKK1 and MKK5 positively regulate the disease resistance against the virulent pathogen, *Pst*DC3000. Figure 6B, C indicates that *mkk1* and *MKK5RNAi* but not *mkk2* plants showed enhanced susceptibility to *F. sporotrichioides*. Thus, the MKD1–MKK1/MKK5 signaling cascade plays positive roles in disease resistance to these pathogens. On the other hand, no lines showed visible phenotypic changes in response to the T-2 toxin compared with the WT (Supplementary Fig. S8). Thus, the phytotoxin response may not be regulated by the MKD1–MKK1/MKK5 pathway.

### MKD1 controls the activation of MPK3 and MPK6 in response to phytotoxin

**Figure 7.**
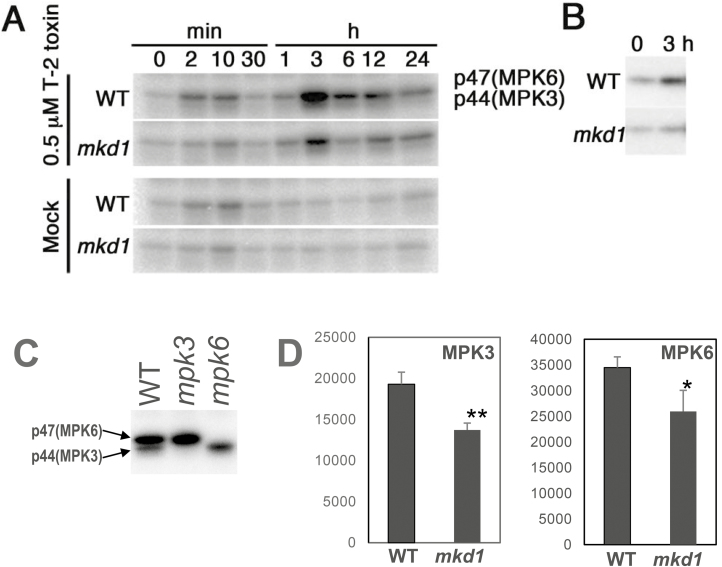
Downstream MAPKs of the MKD1-dependent signaling cascade in response to T-2 toxin. (A) MAP kinase activities were investigated in mock- or T-2 toxin-treated WT and *mkd1* mutant plants by in-gel kinase assays. Similar results were obtained in two independent experiments. (B) Immunoprecipitation kinase assay carried out with T-2 toxin-treated WT and *mkd1* mutant plants using an anti-MPK6 antibody. Similar results were obtained in two independent experiments. (C) p44 and p47 MAPK correspond to MPK3 and MPK6, respectively. MAP kinase activities were examined in WT, *mpk3*, and *mpk6* mutant plants after 3h of T-2 toxin treatment. (D) Activation of MPK3 and MPK6 by T-2 toxin was suppressed in the *mkd1* mutant. These MAPK activities were investigated in WT and *mkd1* mutant plants after 3 h of T-2 toxin treatment by in-gel kinase assays (*n*=3). Then, corresponding bands were quantified. *P<0.05, **P<0.01, based on Student’s *t*-test.

**Figure 8.**
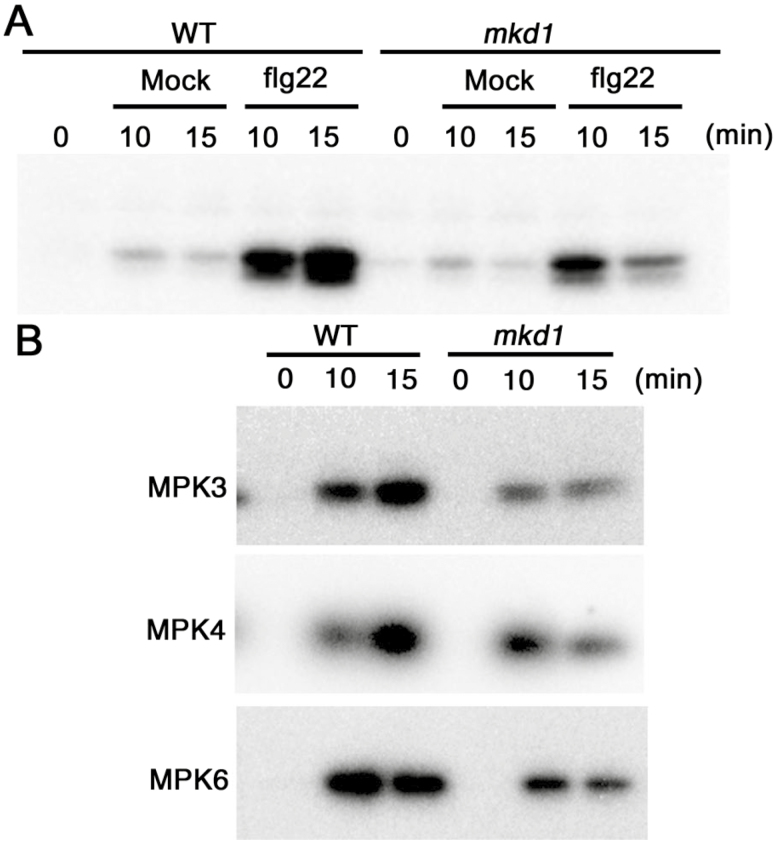
Downstream MAPKs of the MKD1-dependent signaling cascade in response to flg22. (A) MAP kinase activities were investigated in mock- or flg22-treated WT and *mkd1* mutant plants by in-gel kinase assays. Similar results were obtained in two independent experiments. (B) Immunoprecipitation kinase assay carried out with flg22-treated WT and *mkd1* mutant plants using an anti-MPK3, -MPK4, and -MPK6 antibody. Similar results were obtained in two independent experiments.

Of the 20 MAPKs in Arabidopsis, MPK3, MPK4, and MPK6 are known to play important roles in immune responses against phytopathogen infection (Colcombet and Hirt, 2008). Numerous pathogen-derived molecules such as flg22 induce the activities of MPK3 and MPK6 (Colcombet and Hirt, 2008). In addition, the T-2 toxin is a potent activator of MPK3 and MPK6 (Nishiuchi *et al.*, 2006). Therefore, we investigated the effects of MKD1 loss on the activities of these MAPKs in response to the T-2 toxin and flg22. The induction of MPK3 and MPK6 activities by the T-2 toxin and flg22 was significantly decreased by about 30% in the *mkd1* mutant compared with the WT (Figs 7A, B, 8A, B). By contrast, the activation of MPK4 by the T-2 toxin in the *mkd1* mutant was equivalent to that in the WT (see Supplementary Fig. S9). These results suggest that MKD1 controls the activation of MPK3 and MPK6 but not MPK4 in response to the trichothecene phytotoxin. These results suggest that MKD1 is required for the full activation of MPK3 and MPK6 in response to the flg22 and trichothecene phytotoxin. However, the activation of MPK3 and MPK6 by the T-2 toxin was not completely suppressed in the *mkd1* mutant (Fig. 7). The activities of MPK3 and MPK6 are likely regulated by other MAPK signaling pathways as well (Colcombet and Hirt, 2008).

Then, we examined the interactions of MPK3 and MPK6 with MKKs using yeast two-hybrid assays. MPK3 interacted with MKK1, MKK2, and MKK5, whereas MPK6 interacted with MKK2 and MKK5 only (see Supplementary Fig. S10). Correspondingly, the fluorescence signals of transgenic plants expressing GFP-fused MKD1, MKK1, MKK5, and MPK6 were mainly localized in the periphery of root cells (Supplementary Fig. S11). The intracellular localization of MKD1 correlated with that of MKK1, MKK5, and MPK6.

### The phosphorylation of SUMOs, R protein, and GST is decreased in the *mkd1* mutant

A phosphoproteomic analysis was performed to identify target proteins of the MKD1-dependent signaling cascade. Since plant responses to the T-2 toxin were regulated by the MKD1-dependent pathway, we used T-2 toxin-treated samples to identify MKD1 target proteins. Rubisco significantly affects proteome analyses of shoot samples (Aryal *et al.*, 2012). *MKD1* mRNA was strongly expressed in root cells where it was induced by the T-2 toxin (Fig. 2C). Furthermore, T-2 toxin hypersensitivity was observed in the *mkd1* roots (Fig. 3G). Therefore, we performed quantitative phosphoproteomic analyses using root samples. Phosphorylated proteins were purified from roots of *mkd1* mutant and WT plants after T-2 toxin treatment (see Supplementary Fig. S12A, B) and were subjected to iTRAQ proteome analysis (Supplementary Fig. S12A; Jones *et al.*, 2006). The levels of 34 phosphoproteins were 75% lower in the *mkd1* mutant than in the WT (Supplementary Table S3). Interestingly, phosphorylation of the small ubiquitin-related modifier proteins SUMO1 and SUMO2 was significantly decreased in the *mkd1* mutant (Supplementary Table S3). The phosphorylation of the R protein RPP13, glutathione S-transferase9 (GST9), spermidine synthase, and calmodulin also was decreased in the *mkd1* mutant (Supplementary Table S3). These results suggested that the MKD1 cascade positively regulated the phosphorylation of these putative target proteins in response to the phytotoxin. In addition, hyperphosphorylation of HSP90 and the reticulon-like proteins, RTNLB1 and RTNLB2, was observed in the *mkd1* mutant as compared with the WT (Supplementary Table S3). The phosphorylation by alternative kinases of proteins relevant to plant immune responses might be stimulated in the *mkd1* mutant.

## Discussion

Among a large number of MAPKKKs, some MAPKKK-dependent MAPK cascades have been reported to be involved in innate immune responses against phytopathogens (Asai *et al.*, 2002; Hadiarto *et al.*, 2006; Ichimura *et al.*, 2006; Suarez-Rodriguez *et al.*, 2007; Gao *et al.*, 2008; Zhao *et al.*, 2014; Kawasaki *et al.*, 2017; Thulasi Devendrakumar *et al.*, 2018). In this study, we revealed that the MKD1–MKK1/MKK5–MPK3/MPK6–dependent signaling pathway induced by the T-2 toxin and flg22 is involved in disease resistance against fungal and bacterial phytopathogens (Fig. 8). The amino acid sequences of the Raf-like MAPKKKs CTR1 and EDR1 are similar to that of MKD1 (see Supplementary Fig. S2). Although CTR1 belongs to the Raf-like kinase family, its biological function is quite different from that of MKD1. EDR1 negatively regulates disease resistance against *Pst*DC3000 and the powdery mildew fungus (Frye and Innes, 1998; Frye *et al.*, 2001). Thus, MKD1 and EDR1 antagonistically regulate disease resistance against *Pst*DC3000, although the EDR1-depedent MAPK signaling cascade overlaps with the MKD1-dependent pathway. Therefore, our finding of an MKD1-dependent pathway is an important step toward the elucidation of plant MAPK signaling networks that are regulated by defense-related MAPKKKs including the Raf-like MAPKKKs.

Interactions between MKD1 and MKK1 were observed in the nucleus and cytoplasm, while interactions between MKD1 and MKK5 occurred only in the cytoplasm (Fig. 5B). This is consistent with previous reports for MAPKKKs. EDR1 localizes to the endoplasmic reticulum and nucleus (Christiansen *et al.*, 2011) and to the *trans*-Golgi network/early endosome through the action of the KEEP ON GOING (KEG) protein (Gu and Innes, 2011). Similarly, a tobacco MEKK1-like MAPKKK (NPK1) was localized not only to the cytoplasm but also to the nucleus (Nishihama *et al.*, 2001). In addition, MEKK1 directly phosphorylates the transcription factor WRKY53 (Miao *et al.*, 2007). Thus, MAPKKKs localize not only to nuclei but also to other compartments, possibly in response to developmental and/or environmental cues. Using BiFC, Gao *et al.* (2008) showed that MEKK1 interacts with MKK1 and MKK2, suggesting that the downstream MKKs of the MKD1 cascade at least partially overlap with those of MEKK1. In addition, MKD1 interacted with the ANFXL1 protein in the cytoplasm and nucleus (Fig. 1D, E), hinting at the possibility that MKD1 phosphorylates AtNFXL1 directly. However, AtNFXL1 phosphorylation by MKD1 was not observed under our experimental conditions (data not shown).

*In vitro* kinase assays show that MKD1 directly phosphorylates MKK1 and MKK5, but not MKK2. As stated above, the MEKK1–MKK1/MKK2 and EDR1–MKK4/MKK5 pathways are involved in disease resistance against *Pst*DC3000 and *Botrytis cinerea* (Colcombet and Hirt, 2008). We described the MKD1–MKK1/MKK5 pathway as a novel MAP kinase cascade. Furthermore, Ser46 on MKK1 and Thr83, Thr215, and Ser256 on MKK5 were phosphorylated by MKD1 (see Supplementary Table S2). These phosphorylation sites differ from the putative phosphorylation motif of plant MAPKKs, S/TXXXXXS/T (Matsuoka *et al.*, 2002). Matsuoka *et al.* (2002) also suggested that phosphorylation of Thr218 and Thr224 on MKK1 is involved in the activation of MPK4 but not of MPK3. On the other hand, the position of the phosphorylation target Ser27 on MKK1 partially corresponds to the S/TXXXXXS/T motif (Li *et al.*, 2009). These results indicate that the phosphorylation sites of MKK1 may depend on experimental conditions. The phosphorylation site Thr215 on MKK5 also partially corresponds to the S/TXXXXXS/T motif, but Ser83 and Ser256 on MKK5 do not. Ser256 on MKK5 partially corresponds to the SXXXS/T motif, a putative phosphorylation motif of animal MAPKKs (Matsuoka *et al.*, 2002). We suggest that MKD1 directly phosphorylates the serine or threonine residues on MKK1 and MKK5. The BiFC assay using onion epidermal cells indicated a weak interaction between MKD1 and MKK4 (Supplementary Fig. S5). Although other results did not support this possibility (Fig. 5A; Supplementary Fig. S6), the interaction will be checked by BiFC assay using Arabidopsis transgenic plants and genetic analysis of the *mkk4* mutant in the future. MPK3 and MPK6 are known to function downstream of MKK4 and MKK5 (Colcombet and Hirt, 2008), whereas MPK4 is regulated by MKK1 and MKK2 (Colcombet and Hirt, 2008). Genetic analysis has demonstrated the involvement of the MKK1–MPK6 pathway in biotic and abiotic stress responses (Mészáros *et al.*, 2006; Qiu *et al.*, 2008; Xing *et al.*, 2008). However, protein–protein interaction between MKK1 and MPK6 has not been previously shown. The results from the yeast two-hybrid assays suggest the existence of MKK1–MPK3 pathway (Supplementary Fig. S10), which has not been reported before. Therefore, these interactions will be confirmed by further studies in the future. We also revealed that *mpk3* and *mpk6* lack enhanced susceptibility to *F. sporotrichioides* (Supplementary Fig. S13). Similarly, *mpk3* and *mpk6* exhibited normal basal resistance to *Pst*DC3000 and the fungal pathogen *Botrytis cinerea* (Beckers *et al.*, 2009; Galletti *et al.*, 2011). These results likely are due to functional redundancy between the two genes. PAMP-induced resistance against *P. syringae* and *B. cinerea* is positively regulated by MPK3 and MPK6 (Beckers *et al.*, 2009; Galletti *et al.*, 2011). In addition, other MAPKs may also be involved in the MKD1-dependent signaling cascade during immune responses to phytopathogen infection. We suggest a novel MKD1–MKK1/MKK5–MPK3/MPK6 pathway induced by the flg22 and T-2 toxin.

The iTRAQ analysis revealed that SUMO1 and SUMO2 were significantly decreased in the *mkd1* mutant (see Supplementary Table S3). Both proteins are involved in disease resistance against *Pst*DC3000 (van den Burg *et al.*, 2010). In addition, the phosphorylation of disease-resistance (R) proteins is important for signal perception (Martin *et al.*, 2003). We observed MKD1-dependent phosphorylation of the R protein RPP13 (Supplementary Table S3), which may be involved in phytopathogen resistance. Furthermore, since GSTs have been reported to be mycotoxin-detoxifying enzymes (Gardiner *et al.*, 2010), the GST9 phosphorylation found here (Supplementary Table S3) may be related to mycotoxin detoxification in host plants. Phosphorylation of GST proteins by an abiotic stress has also been reported (Chitteti and Peng, 2007). Both spermidine synthase and calmodulin positively regulate disease resistance against phytopathogens (Choi *et al.*, 2009; Nambeesan *et al.*, 2012). Thus, phosphorylation of these proteins by the MKD1-dependent cascade is likely to be involved in phytotoxin responses and disease resistance against phytopathogens. In addition, hyperphosphorylation of some defense-related proteins was observed in the *mkd1* mutant (Supplementary Table S3). The HSP90 chaperone complex is required for the R protein-mediated defense response to pathogens (Sangster and Queitsch, 2005). Phosphorylation of HSP90 attenuates interactions with co-chaperones (Mollapour and Neckers, 2012). RTNLB1 regulates the activity of the flagellin-sensitive 2 (FLS2) immune receptor (Lee *et al.*, 2011). Phosphorylation of these innate immune-related proteins may be negatively regulated by MKD1-dependent signaling cascades. Alternatively, the loss of MKD1 function may affect the MAPK signaling network, resulting in hyperactivation of other MAPK cascades. Our identification of an MKD1-dependent pathway opens the door for the elucidation of MAPK signaling networks regulated by Raf-like MAPKKKs.

## Supplementary data

Supplementary data are available at *JXB* online.

Fig. S1. Interaction of MKD1 with AtNFXL1 studied by BiFC analysis.

Fig. S2. Structure and amino acid sequence of the MKD1 protein.

Fig. S3. GUS staining of PMKD1:GUS plants.

Fig. S4. Expression pattern of *PR1* and *PDF1.2* in the *mkd1* mutant.

Fig. S5. BiFC assays between MKD1 and MKK1, MKK2, MKK4, MKK5 using onion epidermis.

Fig. S6. Phosphorylation of MKK1, MKK2, and MKK4 by constitutively active MKD1 (ΔMKD1) was investigated by *in vitro* kinase assays.

Fig. S7. RT-PCR of *MKK1* and *MKK2* mRNA in *mkk1* and *mkk2* mutants.

Fig. S8. WT, *mkk1*, and *mkk2* mutants, and *MKK5RNAi* transgenic plants grown on MS agar medium with or without 0.5 μM T-2 toxin.

Fig. S9. Immunoprecipitation kinase assay carried out using an anti-MPK4 antibody in T-2 toxin-treated WT and *mkd1* mutant plants.

Fig. S10. Downstream MPKs of the MKD1-dependent pathway.

Fig. S11. Subcellular localization of MKD1, MKK1, MKK5, and MPK6 in root cells of Arabidopsis.

Fig. S12. Inoculation assays in *mpk3* and *mpk6* mutants using *F. sporotrichioides*.

Fig. S13. Quantitative phosphoproteomic analysis using iTRAQ and the Pro-Q^®^ Diamond Enrichment Kit.

Table S1. Primers used in this study.

Table S2. Phosphorylation sites of MKK1 and MKK5 targeted by MKD1.

Table S3. Phosphoproteomic analysis using roots of T-2 toxin-treated *mkd1* mutants.

erz556_suppl_supplementary_figures_S1-S13

erz556_suppl_supplementary_tables_S1-S3
