# Supplementary material for: Arabidopsis MAPKKK δ-1 is required for full immunity against bacterial and fungal infection
Source: J Exp Bot. 2019 Dec 17;71(6):2085–97. doi: 10.1093/jxb/erz556 (PMC7094076; doi:10.1093/jxb/erz556)
Supplement: erz556_suppl_supplementary_figures_S1-S13 [file erz556_suppl_supplementary_figures_s1-s13.pdf]

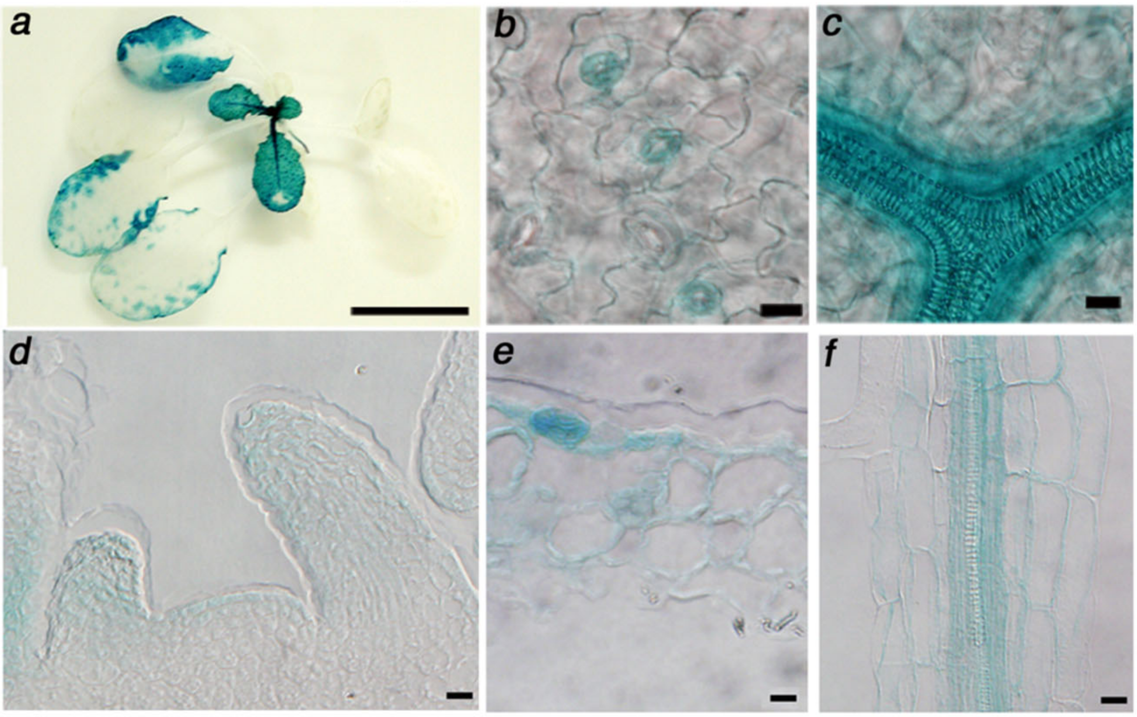

Supplementary Figure 1. GUS staining of PMKD1:GUS plants. Representative photograph of a 25-days-old plant (*a*). Nomarski images of epidermal cells (*b*), vascular bundle (*c*), vertical section of shoot apex (*d*), rosette leaf (*e*) and root (*f*). Scale bars: 1 cm (*a*); 100 µm (*b* and *c*); 10 µm (*d*-*f*).

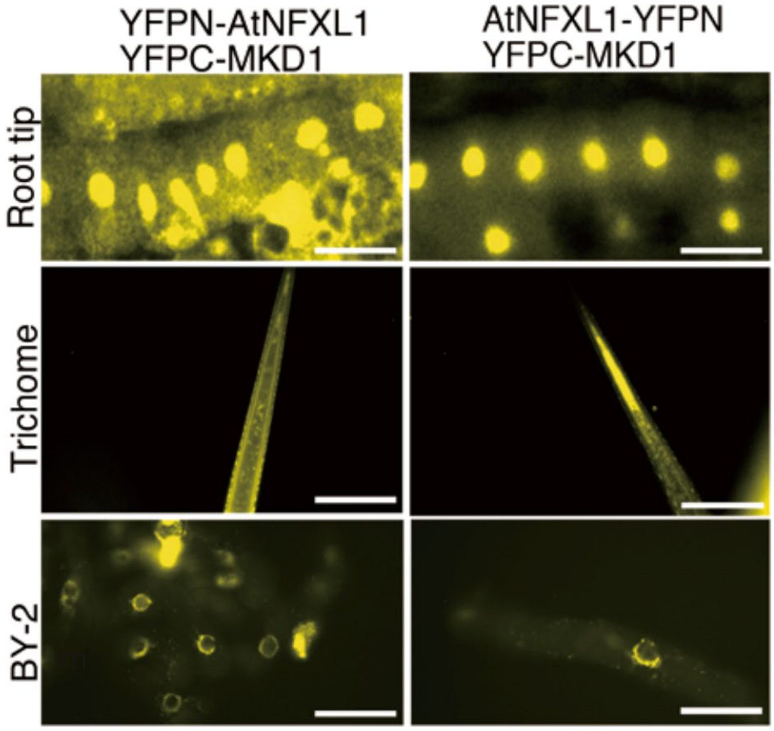

Supplementary Figure 2. The interaction analysis of MKD1 with AtNFXL1 by BiFC method. Fluorescence signals were observed in root tip and trichome in Arabidopsis cells and tobacco BY-2 cells. Scale bars = 10 µm (upper panels) . Scale bars = 100 µm (middle and lower panels).

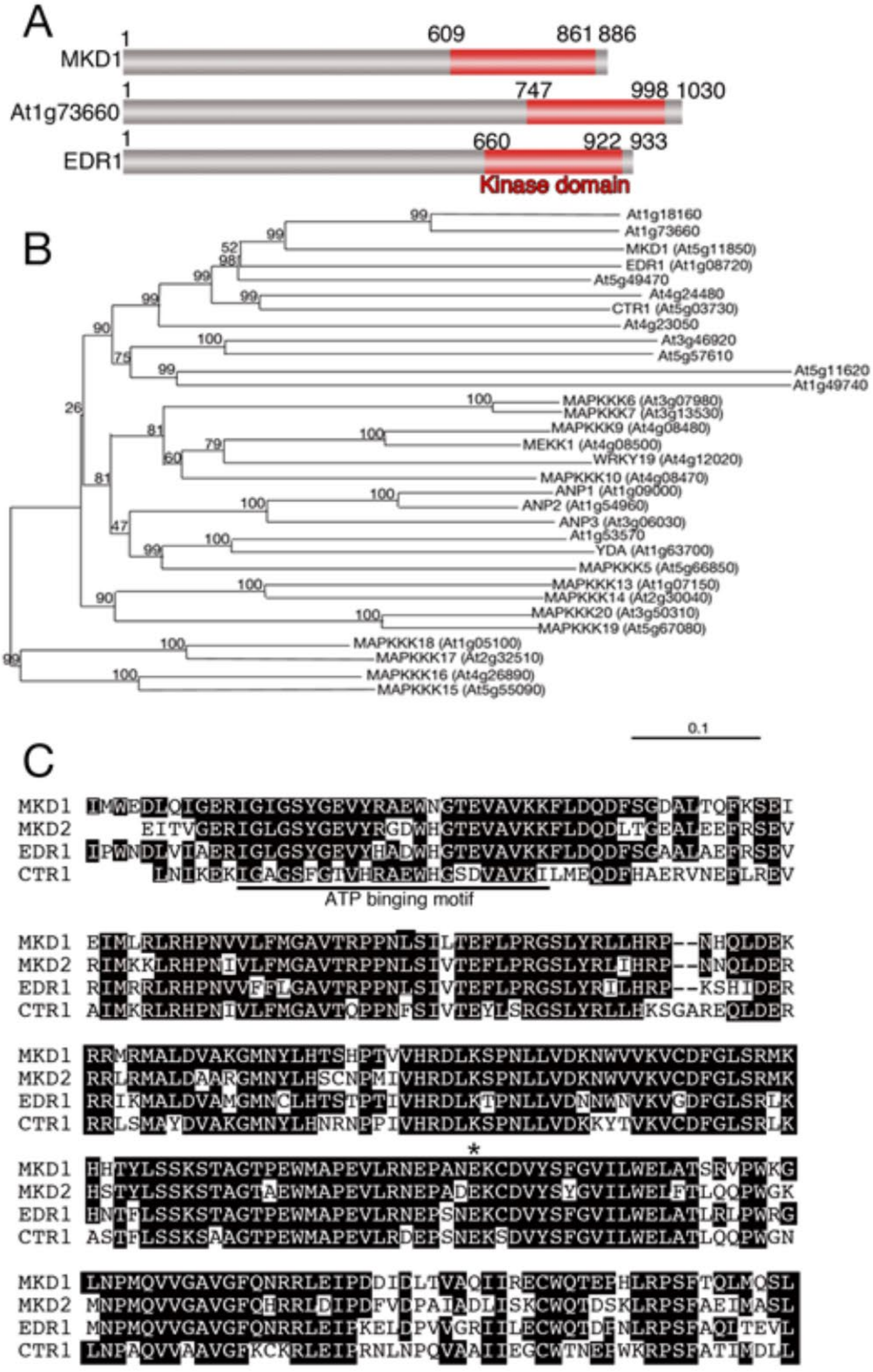

Supplementary Figure 3. Schematic structure and amino acid sequence of MKDl protein. (A) Schematic structure of MKD1 and homologue proteins. (B) The phylogenetic tree of MAPKKKs in Arabidopsis. (C) Alignments of amino acid sequences around the kinase domain MKD1, MKD 2, EDR1 and CTR1 protein. Under line shows ATP binging motif. Asterisk shows putative T-DNA insertion position in the *mkd1* mutant.

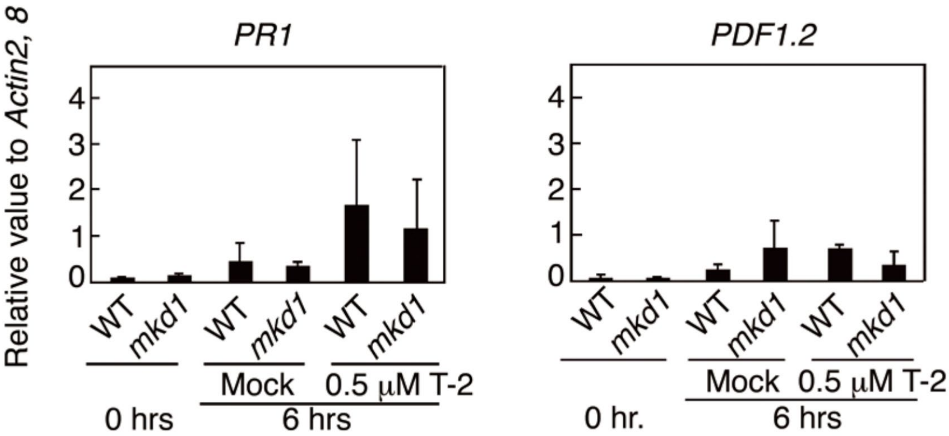

Supplementary Figure 4. The expression pattern of *PR1* and *PDF1.2* in the *mkd1* mutant. WT and mkd1 mutant plants were grown on MS medium for 10 days, then, transferred to MS agar plates with or without T-2 toxin. The expression level of both gene are not significantly different between WT and the *mkd1* mutant. Total RNAs were prepared in T-2 toxin-treated WT and *mkd1* mutant for 6 hours.

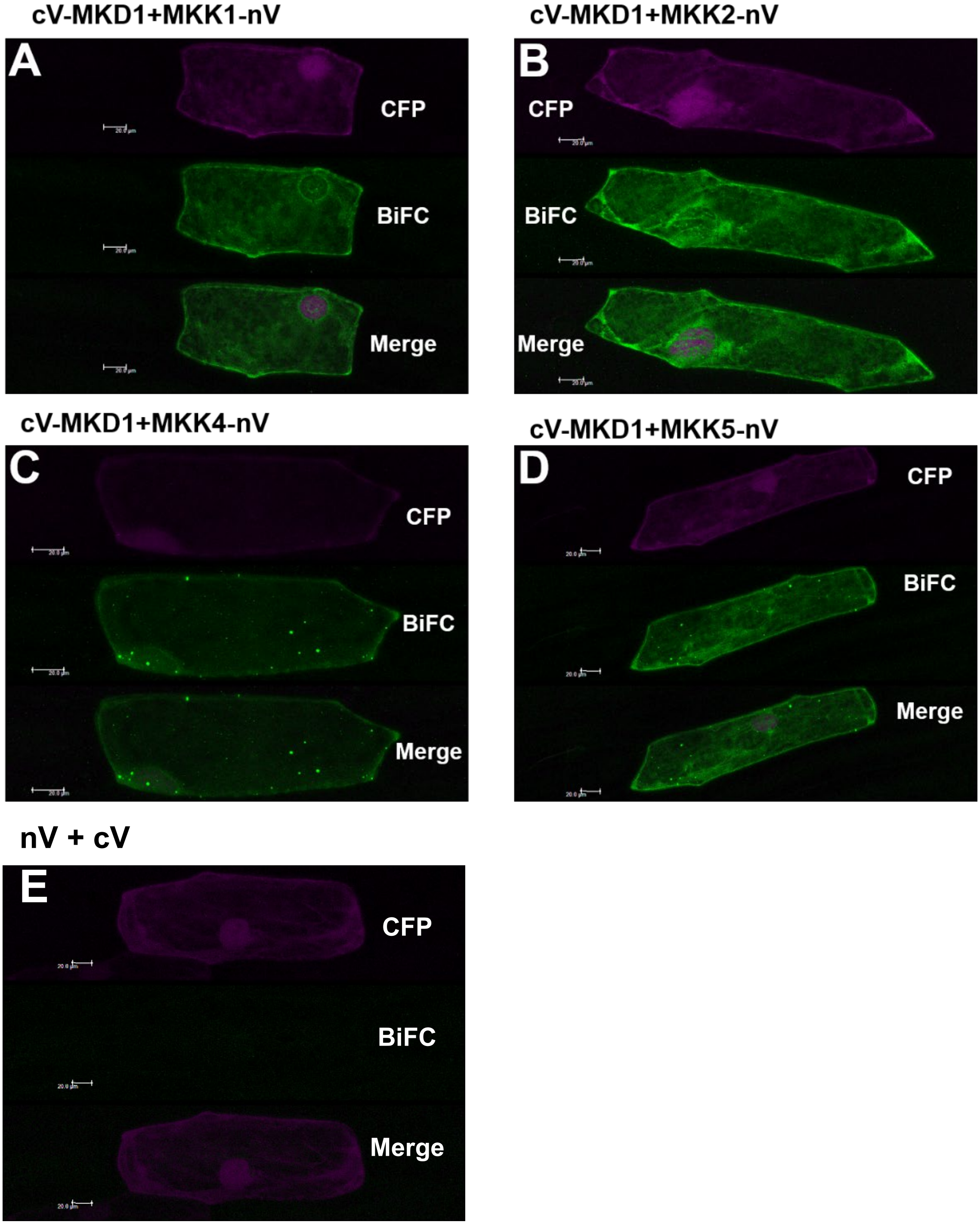

Supplementary Figure 5. BiFC assays between MKD1 and MKK1, MKK2, MKK4, MKK5 using onion epidermis (A-D). (E) nV + cV: negative control. The CFP signals show internal control. Green fluorescence signals were shown as BiFC signals. Scale bars = 20 μm.

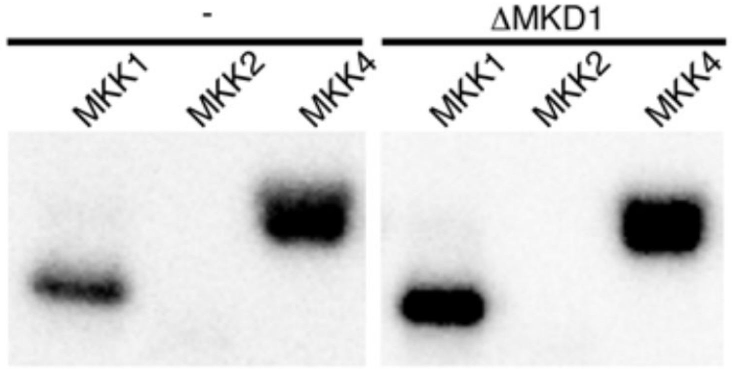

Supplementary Figure 6. The Phosphorylations of MKK1, MKK2, and MKK4 by constitutively active MKD1 (ΔMKD1) were investigated by *in vitro* kinase assays.

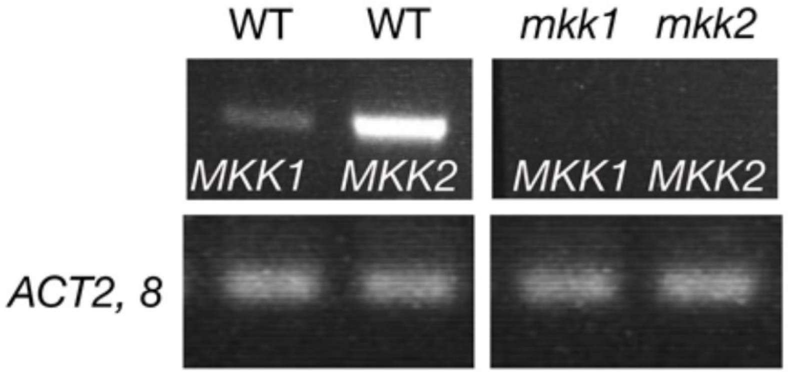

Supplementary Figure 7. RT- PCR analysis of *MKK1* and *MKK2* mRNA in the *mkkl* and *mkk2* mutant. *ACT2, 8* was used as reference genes.

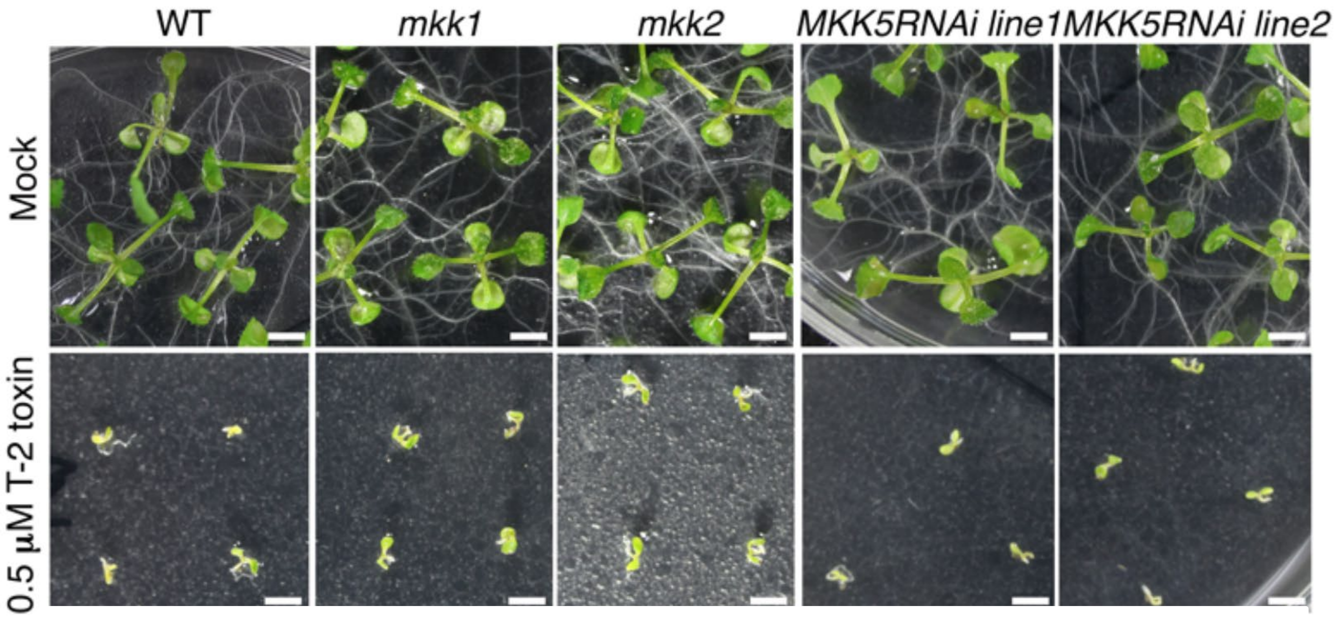

Supplementary Figure 8. WT, *mkkl*, and *mkk2* mutant and *MKK5RNAi* transgenic plants were grown on MS agar medium with or without 0.5 µM T-2 toxin for two weeks. Scale bars: 1 mm.

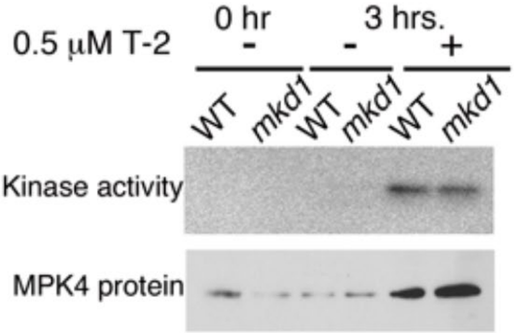

Supplementary Figure 9. The Immunoprecipitation kinase assay was carried out using an anti-MPK4 antibody in WT and *mkd1* mutant plants with or without T-2 toxin.

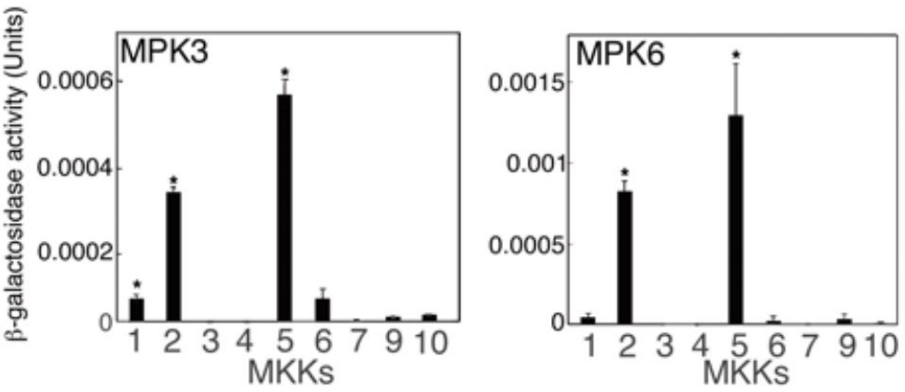

Supplementary Figure 10. Downstream MPKs of the MKD1-dependent pathway. Protein-protein interactions between MPKs and MKKs were as assessed by yeast 2-hybrid analysis; Strength of protein interactions are shown by the β-galactosidase activity units per number of cells and incubation time. Data represent the mean ± s.d. of 3 replicate experiments. Asterisks indicate the statistical significance at $P < 0.05$.

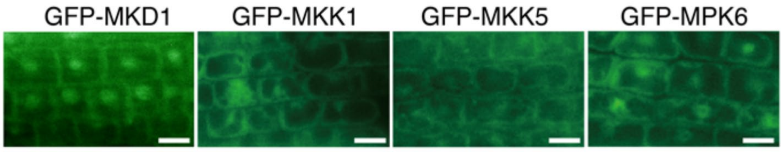

Supplementary Figure 11. The subcellular localization of MKD1, MKK1, MKK5 and MPK6 in root cells of *Arabidopsis*. The fluorescence in root cells of transgenic plant expressing GFP-MKD1, GFP-MKK1, GFP-MKK5 and GFP-MPK6 protein. Scale bars = 10μm.

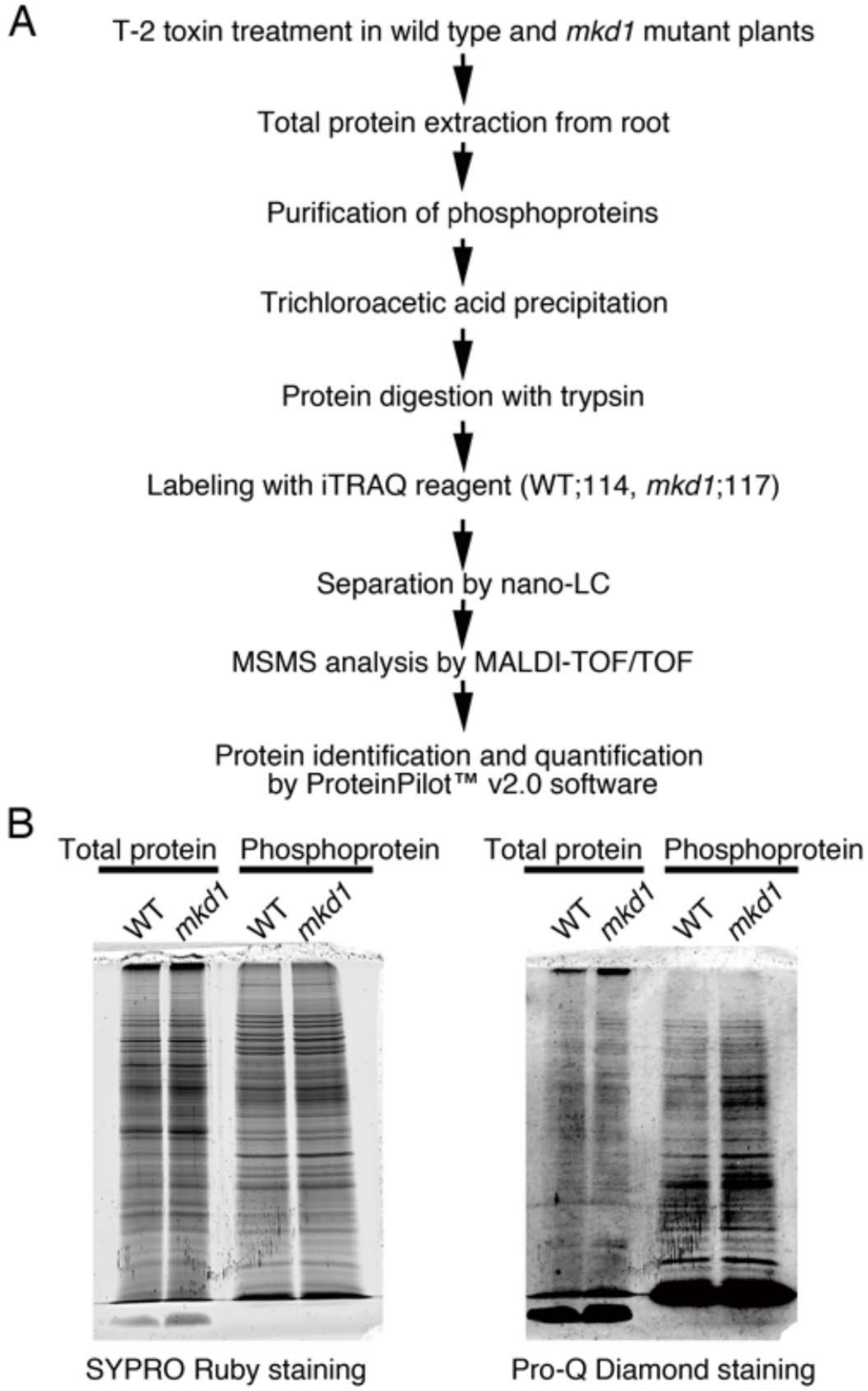

Supplementalry Figure 12. Scheme of quantitative phospho-proteomic analysis using iTRAQ and Pro-Q® Diamond Enrichment Kit. (A) Schematic view of the work flow in a quantitative phosphoproteomic analysis. (B) Total proteins and phosphoproteins were stained by SYPRO® Ruby and Pro-Q® Diamond. The each proteins were loaded 10μg.

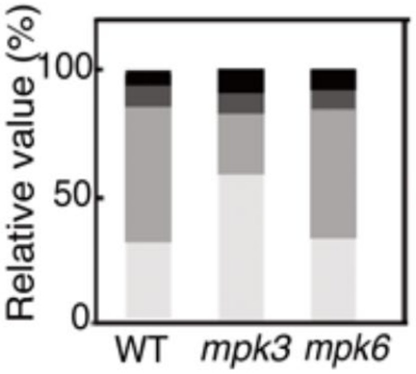

Supplementary Figure 13. The inoculation assays in the WT, *mpk3* and *mpk6* mutant plants using *F. sporotrichioides*. The relative values of the disease symptoms were classified as follows: White (class1); normal. Grey (class2); changed to black leaf. Dark grey (class3) ; partial hyphae. Black (class4); expanded aerial hyphae.
