# Supplementary material for: Arabidopsis MAPKKK δ-1 is required for full immunity against bacterial and fungal infection
Source: J Exp Bot. 2019 Dec 17;71(6):2085–97. doi: 10.1093/jxb/erz556 (PMC7094076; doi:10.1093/jxb/erz556)
Supplement: erz556_suppl_supplementary_tables_S1-S3 [file erz556_suppl_supplementary_tables_s1-s3.pdf]

Supplementary Table 1. The prims used in this study

Name	Sequence (5' – 3')	Application
MKD1F-1	GCGAATTCATGGGTGGCCCCGTTGAAA	Yeast two-hybrid analysis
MKD1F-2	GCCTCGAGGTTTGTCTCCTCTCGTCTGA	Yeast two-hybrid analysis, Complementation analysis
MKD1F-3	GCGAATTCATGGGTGGCCCCGTTGAAA	Protein synthesis
MKD1F-4	GCGTCGACGTTTGTCTCCTCTCGTCTGA	Protein synthesis
MKD1F-5	CACCATGGGTGGCCCCGTTGAAA	BiFC analysis
MKD1F-6	CATCAAACTTTCACTCGTATTC	BiFC analysis
MKD1F-7	GCCCCGGGCATGGGTGGCCCCGTTGAAA	GFP analysis
MKD1F-8	GCGCGGCCGCTTACGTTTGCCAACATTCACG	GFP analysis
MKD1-9	CACCGCATTGAGAAGGTGGATGCT	Northern blot analysis
MKD1-10	AACCTCAGAAGGGATCAGAG	Northern blot analysis
MKD1-11	CTGGAAAGGTTTGAACCCGA	Northern blot analysis
MKD1-12	CTGGAAAGGTTTGAACCCGA	Northern blot analysis
MKD1KD-1	GCGCGATCGCATGTGGGAAGATCTTCAGATTG	Protein synthesis
MKD1KD-2	GTTTAAACTCAGTGGTGGTGGTGGTGGTGGCTTATGTTTAGACCCTG	Protein synthesis
MKD1KD-3	GCCCCGGGGATGTGGGAAGATCTTCAGATTG	Yeast two-hybrid analysis
MKD1KD-4	GCCTCGAGTTAGCTTATGTTTAGACCCTG	Yeast two-hybrid analysis
MKD1P-1	GCAAGCTTCACAAGAGCAGAGGTTGGAG	GUS analysis, Complementation analysis
MKD1P-2	GCTCTAGATACCAGCATACATGGGAGAT	GUS analysis
MKK1F-1	GCCCCGGGGATGAACAGAGGAAGCTTATGC	Yeast two-hybrid analysis, Protein synthesis
MKK1F-2	GCCTGCAGTAGTTAGCAAGTGGGGGAA	Yeast two-hybrid analysis, Protein synthesis
MKK2F-1	GCCCCGGGGATGAAGAAAGGTGGATTCAGC	Yeast two-hybrid analysis, Protein synthesis
MKK2F-2	GCCTGCAGTTACACGGAGAACGTACCAGAC	Yeast two-hybrid analysis, Protein synthesis
MKK3F-1	GCCCCGGGGATGGCGGCATTGGAGGAGC	Yeast two-hybrid analysis
MKK3F-2	GCCTGCAGCTAATCTAAGTTTGTAATATAAAGC	Yeast two-hybrid analysis
MKK4F-1	GCCCCGGGGATGAGACCGATTCAATCGCC	Yeast two-hybrid analysis
MKK4F-2	GCCTGCAGCTATGTGGTTGGAGAAGAAGA	Yeast two-hybrid analysis
MKK5F-1	GCCCCGGGGATGAAACCGATTCAATCTCCTT	Yeast two-hybrid analysis, Protein synthesis
MKK5F-2	GCCTGCAGCTAAGAGGCAGAAGGAAGAG	Yeast two-hybrid analysis, Protein synthesis
MKK6F-1	GCCCCGGGGATGGTGAAGATCAAATCGAACT	Yeast two-hybrid analysis
MKK6F-2	GCCTGCAGTTATCTAAGGTAGTTAACAGGTGG	Yeast two-hybrid analysis
MKK7F-1	GCCCCGGGGATGGCTCTTGTTCGTAAACGC	Yeast two-hybrid analysis
MKK7F-2	GCCTGCAGCTAAAGACTTTCACGGAGAAAAG	Yeast two-hybrid analysis
MKK9F-1	GCCCCGGGGATGGCTTTAGTACGTGAACGT	Yeast two-hybrid analysis
MKK9F-2	GCCTGCAGTCAAAGATCTTCCCGGAGAAA	Yeast two-hybrid analysis
MKK10F-1	GCCCCGGGGATGACACTTGTTAGAGAACGAC	Yeast two-hybrid analysis
MKK10F-2	GCCTGCAGCTATCTGTTTTTCACAAAAGAAT	Yeast two-hybrid analysis
MKK1F-3	CACCATGAACAGAGGAAGCTTATGC	BiFC analysis
MKK1F-4	TAGTTAGCAAGTGGGGGAA	BiFC analysis
MKK2F-3	CACCATGAAGAAAGGTGGATTCAGC	BiFC analysis
MKK2F-4	TTACACGGAGAACGTACCAGAC	BiFC analysis
MKK5F-3	CACCATGAAACCGATTCAATCTCCTT	BiFC analysis
MKK5F-4	CTAAGAGGCAGAAGGAAGAG	BiFC analysis
MKK5RNAiF-1	CACCGCTTCACCTATGAAGAACCG	MKK5RNAi
MKK5RNAiF-2	TCACTCGTTCTAGCTCGGAT	MKK5RNAi
MPK3F-1	GCCCCGGGTATGAACACCGGCGGTGG	Yeast two-hybrid analysis
MPK3F-2	GCGTCGACCTAACCGTATGTTGGATTGAGT	Yeast two-hybrid analysis
MPK6F-1	GCCCCGGGTATGGACGGTGGTTCAGGTC	Yeast two-hybrid analysis
MPK6F-2	GCGTCGACCTATTGCTGATATTCTGGATTGA	Yeast two-hybrid analysis
AtNFXL1F-3	CACCATGAGCTTTCAAGTCAGGCG	BiFC analysis
AtNFXL1F-4	CTCACATACCTTCTCCCAGT	BiFC analysis
AtNFXL1F-1	CGCCAT ATGAGCTTTCAAGTCAGGCG	Yeast two-hybrid analysis
AtNFXL1F-2	GACGTCGACCTCACATACCTTCTCCCAGT	Yeast two-hybrid analysis
AtNFXL1ΔC-1	CGCCAT ATGAGCTTTCAAGTCAGGCG	Yeast two-hybrid analysis
AtNFXL1ΔC-2	GACGTCGACTAGCGGTTATACGACCACATG	Yeast two-hybrid analysis
AtNFXL1ΔN-1	CGCCATATGAAGGAGGAGAATGTTGTGTTG	Yeast two-hybrid analysis
AtNFXL1ΔN-2	GACGTCGACCTCACATACCTTCTCCCAGT	Yeast two-hybrid analysis
AtNFXL1ΔNΔZn-1	CGCCATATGCATGTGGTCGTATAACCGCTA	Yeast two-hybrid analysis, Protein preparation
AtNFXL1ΔNΔZn-2	GACGTCGACCTCACATACCTTCTCCCAGT	Yeast two-hybrid analysis, Protein preparation
MKK1 F-5	CACCATGAACAGAGGAAGCTTATGC	GFP analysis
MKK1 F-6	TAGTTAGCAAGTGGGGGAA	GFP analysis
MKK5 F-5	CACCATGAAACCGATTCAATCTCCTT	GFP analysis
MKK5F-6	CTAAGAGGCAGAAGGAAGAG	GFP analysis
MPK6 F-3	CACCATGGACGGTGGTTCAGGTC	GFP analysis
MPK6 F-4	CTATTGCTGATATTCTGGATTGA	GFP analysis
PR1F1	ATTACTTCATTAGTATGGCTTCT	RT-qPCR
PR1F2	CTTGTCTGGCGTCTCC	RT-qPCR
ACT2/8 F1	CATCACACTTTCTACAATGAGCT	RT-qPCR
ACT2/8 F2	CGACCTTAATCTTCATGCTGC	RT-qPCR
PDF1.2F1	CACCCTTATCTTCGCTGCTC	RT-qPCR
PDF1.2F2	GTTGCATGATCCATGTTTGG	RT-qPCR

Supplementary Table 2. The phosphorylation sites of MKK1 and MKK5 by MKD1

	Sequence	Modifications	ΔMass	Prec MW	Sc
MKK1 (S46)	QTVSLSEPGAPPPIEPL	Phospho(S)@4; Oxidation(P)@13	0.0056	1826.8759	6
	QTVSLSEPGAPPPIEPLDN	Oxidation(P)@13; Deamidated(N)@19	-0.1838	1976.774	9
	QTVSLSEPGAPPPIEPL		0.0096	1730.9186	4
	SEPGAPPPIEPL	Oxidation(P)@8	-0.178	1218.4352	4
	KDGDLRVNKDGIQTVSLSEPGAPPPIEPL		-0.304	3041.2993	11
	KDGDLRVNKDGIQTVSLSEPGAPPPIEPLDNQL		-0.3072	3511.5085	18
	KDGDLRVNKDGIQTVSLSEPGAPPPIEPLDNQL		-0.2389	3511.5769	12
MKK5 (T83)	SELERVNRIGSGAGGTVY	Phospho(T)@16	-0.131	1943.7792	5
	SELERVNRIGSGAGGTVY		-0.1321	1863.8118	12
	GSGAGGTVY		0.1133	767.4583	3
	GSGAGGTVY		0.1219	767.4669	4
	GSGAGGTVY		0.1186	767.4636	3
	GSGAGGTVY		0.1343	767.4792	3
	GSGAGGTVY		0.139	767.484	4
	GSGAGGTVY		0.1388	767.4838	3
MKK5 (T215)	GVSRILAQTMDPCNSSVGTIAY	Phospho(T)@9; Carbamidomethyl(C)@13; Methyl(N)@14; Phospho(T)@19	-0.1496	2512.9236	9
MKK5 (S256)	AGDVWSLGVSIL	Trp->Met@5; Phospho(S)@10	-0.0317	1240.5457	8
	NHGRYDGYAGDVWSLGVSILEFYL	Deamidated(R)@4	-0.1917	2731.1001	3
	SLGVSILEF		-0.063	963.4647	14
	SLGVSILEF		-0.0612	963.4666	14
	SLGVSILEF		-0.0539	963.4738	14
	SLGVSILEF		-0.0697	963.458	13
	SLGVSILEFY		-0.0834	1126.5076	15
	SLGVSILEFY		-0.0766	1126.5144	16
	SLGVSILEFY		-0.0877	1126.5033	16
	SLGVSILEFY		-0.086	1126.505	16

Sc shows the score that is a count of the MS/MS peaks that match to a theoretical ion, for those ion types considered by the Paragon™ Algorithm.

Supplementary Table 3. The phosphoproteomic analysis using root in T-2 toxin-treated *mkd1* mutant

mkd1 /WT	Description	AGI	% Cov.	Total
0.30	Maternal effect embryo arrest 58 (MEE58)	AT4G13940	1.6	2.0
0.48	Copper chaperone (CCH)	AT3G56240	57.9	4.2
0.54	Small ubiquitin-like modifier 2 (SUMO2)	AT5G55160	30.1	6.0
0.58	NADH-ubiquinone oxidoreductase-related	AT5G52840	16.0	2.0
0.59	Cobalamin-independent methionine synthase*	AT5G17920	24.8	12
0.62	Putative heat-shock protein	AT3G07770	13.1	7.8
0.63	Small ubiquitin-like modifier 1 (SUMO1)	AT4G26840	30.8	6.0
0.64	Unknown protein	AT1G13930	39.4	6.7
0.65	Malate dehydrogenase	AT1G04410	35.2	16
0.68	Calmodulin 1 (CAM1)	AT5G37780	68.3	18
0.68	Strong similarity to 60S ribosomal protein L17 (gb|X01694)	AT3G04400	11.5	4.0
0.68	Spermidine synthase 1 (SPDS1)	AT1G23820	12.2	6.0
0.69	Glycine-rich protein 2*	AT4G38680	37.7	2.4
0.69	Glutamine synthetase, putative	AT1G66200	5.9	3.5
0.70	Translationally controlled tumor protein (TCTP)	AT3G16640	58.3	16
0.70	Pentatricopeptide repeat-containing protein	AT1G12300	11.7	11
0.71	Glycine-rich RNA binding protein	AT2G21660	45.1	2.0
0.71	Nuclear RNA binding protein A-like protein *	AT5G47210	39.2	6.7
0.71	PDI-LIKE 2-3 (ATPDIL2-3)	AT2G32920	4.3	2.9
0.71	Unknown protein*	AT2G32920	3.3	2.1
0.71	S-adenosylmethionine synthetase	AT1G02500	42.1	15
0.71	ATNADP-ME3 (NADP-malic enzyme 3)	AT5G25880	9.4	2.1
0.72	TRF-LIKE 5 (TRFL5)	AT1G15720	4.9	2.0
0.72	Early-responsive to dehydration 2 (ERD2)*	AT1G56410	30.3	33
0.72	NADH-dependent glutamate synthase*	AT5G53460	7.8	3.3
0.72	Vacuolar calcium-binding protein-like protein	AT1G12080	36.2	4.0
0.73	Heat shock factor binding protein	AT4G15802	14.0	2.0
0.73	Disease resistance protein RPP13 variant	AT3G46530	7.1	2.1
0.74	Glutathione s-transferase phi 9 (ATGSTF9)	AT2G30860	30.7	4.0
0.74	Putative 2,3-bisphosphoglycerate-independent phosphoglycerate mutase	AT1G09780	13.8	5.7
0.74	ATPases associated with various cellular activities	AT1G03000	3.3	2.1
0.74	Flavonol synthase 5 (FLS5)*	AT5G63600	5.2	2.0
0.75	Embryo defective 2296 (EMB2296)*	AT2G18020	54.7	21
0.75	Hydrogen ion transporting ATP synthase	AT1G20260	25.5	9.3
1.32	Nucleosome assemly protein1;1 (NAP1;1)	AT4G26110	40.9	14
1.35	Reticulan like protein B1 (RTNLB1)	AT4G23630	20.7	3.8
1.35	Reticulan like protein B3 (RTNLB3)	AT1G64090	7.8	4.0
1.37	Putative heat shock protein 81-2 (HSP81-2)	At5g56030	57.4	75
1.45	Similar to acidic ribosomal protein p1	AT4G00810	59.1	5.9
1.50	Ubiquitin extension protein, putative / 40S ribosomal protein S27A (RPS27aA	AT1G23410	46.2	11
1.50	Tubulin alpha-3	AT5G1977	32.4	20
1.68	Glyceraldehyde-3-phosphate dehydrogenase of plastid (GAPCP-1)	AT1G79530	21.8	4.2
1.73	60S acidic ribosomal protein P1-like protein	AT5G47700	57.5	5.9
2.60	Dehydrin family protein	AT1G54410	18.4	2.0
4.10	Forkhead-associated domain-containing protein / FHA domain-containing prot	AT5G47790	4.1	2.0
5.07	Heat shock protein 90A *	AT5G52640	34.9	28
11.2	Heat shock protein 70B (Hsp70b)*	AT1G16030	25.7	16

The ratio of *mkd1* /WT shows the fold-change in phosphoprotein expression between WT and *mkd* 1 mutant after T-2 toxin treatment. Data are mean of 3 independent experiments.

These phosphoproteins were statistically significant (P<0.05) and <0.75 or >1.33 fold change in expression between the *mkd* 1 mutant and WT. % Cov (Coverage) shows the percentage of
matching amino acids from identified peptides having Confidence greater than 0 divided by the total number of amino acids in the sequence. Total (ProtScore) shows a measure of the total amount of evidence for a detected protein. Total ProtScore is calculated using all of the peptides detected for the protein. Asterisks show the proteins that was detected the phosphopeptide.
